# Prognostic and Clinicopathological Significance of Epidermal Growth Factor Receptor (EGFR) Expression in Oral Squamous Cell Carcinoma: Systematic Review and Meta-Analysis

**DOI:** 10.3390/ijms241511888

**Published:** 2023-07-25

**Authors:** José Luis Cívico-Ortega, Isabel González-Ruiz, Pablo Ramos-García, David Cruz-Granados, Valerie Samayoa-Descamps, Miguel Ángel González-Moles

**Affiliations:** 1 School of Dentistry, University of Granada, 18071 Granada, Spain; josecivico88@correo.ugr.es (J.L.C.-O.); dacruzgr@correo.ugr.es (D.C.-G.); vasamayoa@correo.ugr.es (V.S.-D.); 2 Instituto de Investigación Biosanitaria ibs.GRANADA, 18012 Granada, Spain; 3 Hospital Universitario San Juan de Reus, CAP Marià Fortuny, 43204 Tarragona, Spain; isagonzru@gmail.com

**Keywords:** epidermal growth factor receptor, EGFR, oral cancer, prognosis, systematic review, meta-analysis

## Abstract

The aim of this systematic review and meta-analysis was to evaluate the current evidence in relation to the clinicopathological and prognostic significance of epidermal growth factor receptor (EGFR) overexpression in patients with oral squamous cell carcinoma (OSCC). We searched MEDLINE/PubMed, Embase, Web of Science, and Scopus for studies published before November 2022. We evaluated the quality of primary-level studies using the QUIPS tool, conducted meta-analyses, examined inter-study heterogeneity via subgroup analyses and meta-regressions, and performed small-study effects analyses. Fifty primary-level studies (4631 patients) met the inclusion criteria. EGFR overexpression was significantly associated with poor overall survival (hazard ratio [HR] = 1.38, 95% confidence intervals [CI] = 1.06–1.79, *p* = 0.02), N+ status (odds ratio [OR] = 1.37, 95%CI = 1.01–1.86, *p* = 0.04), and moderately–poorly differentiated OSCC (OR = 1.43, 95% CI = 1.05–1.94, *p* = 0.02). In addition, better results were obtained by the application of a cutoff point ≥10% tumor cells with EGFR overexpression (*p* < 0.001). In conclusion, our systematic review and meta-analysis supports that the immunohistochemical assessment of EGFR overexpression may be useful as a prognostic biomarker for OSCC.

## 1. Introduction

The global incidence of oral cancer is estimated to be 377,713 new cases per year, resulting in approximately 177,757 deaths, according to recent statistics from GLOBOCAN (IARC, WHO) [1]. Among all oral malignancies, oral squamous cell carcinoma (OSCC) accounts for approximately 90%, with a 5-year mortality rate approaching 50%. In the year 2000, Hanahan and Weinberg [2] introduced a set of distinctive characteristics that malignant neoplastic cells are expected to possess, irrespective of the tissue of tumor origin. This proposal has been designated as hallmarks of cancer, and was later updated and improved with new characteristics [3], which overall consist of sustaining proliferative signaling, evading growth suppressors, resistance to cell death, enabling replicative immortality, angiogenesis, activating invasion and metastasis, deregulating cellular energetics, and avoiding immune destruction, as well as two enabling characteristics, genome instability and mutation and tumor-promoting inflammation. The proposal of cancer hallmarks has had an enormous impact and influence on the scientific community and on the development of emerging lines of research in order to evaluate biomarkers in different cancers for diagnostic, prognostic, or treatment purposes. It should be noted, however, that limited evidence on the implications of these distinctive features in oral cancer is available to date [4].

Among these hallmarks of cancer, the ability of sustaining proliferative signaling is of remarkable relevance in oral oncogenesis [2,3], and in this regard, the epidermal growth factor receptor (EGFR) has received singular attention, having been extensively studied [5]. EGFR, also known as ErbB1/HER1, is the prototypical receptor of the EGFR tyrosine kinase receptors family, which also includes ErbB2/HER2/Neu, ErbB3/HER3, and ErbB4/HER4 [6]. The most recognized ligand of EGFR is EGF, although the receptor can also be activated by ligands such as TGF-α or HB-EGF, among others [7]. The preceding ligands appear to activate EGFR by the same mechanism of ligand binding, with receptor dimerization and recruitment of other signaling proteins [8]. The receptor can be constitutively activated by gene amplification or mutations, leading to a complex EGFR-mediated signal transduction with regulation of downstream molecular signaling pathways, being the most relevant MAPK (ras-Raf-MEK-Erk) and PI3K (PI3k-Akt-mTor) [9]. These pathways culminate in cell proliferation stimulating actions through the activation of transcription factors with upregulation of important oncogenes (*CCND1*/cyclin D1) among the most relevant [10,11]). EGFR overexpression has been considered a poor prognostic factor in cancers of the head and neck, esophagus, ovary, uterine cervix, and bladder through primary-level studies [12,13,14,15,16]. Moreover, the relevant oncogenic implications of EGFR have justified its consideration as a molecular target, cetuximab being the first monoclonal antibody to be approved by the FDA for the treatment of head and neck cancer [17].

Despite the relevance of EGFR, it seems surprising that there are no published evidence-based results through systematic reviews and meta-analyses specifically designed for oral cancer to date. Therefore, based on this background, our aim was to qualitatively and quantitatively evaluate the prognostic and clinicopathologic implications of EGFR overexpression in patients with OSCC.

## 2. Results

### 2.1. Results of the Literature Search

**Figure 1:**
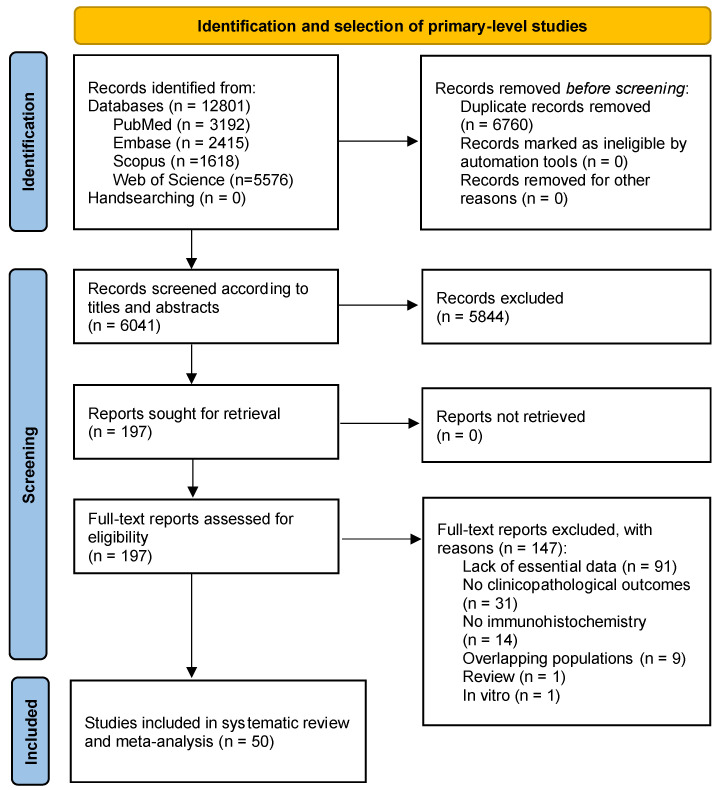
Flow diagram showing the identification and selection process of relevant studies, analyzing the prognostic and clinicopathological significance of EGFR overexpression in OSCC.

The flow diagram in Figure 1 depicts the process of identification, screening, and selection of primary-level studies. A total of 12,801 records were retrieved: 2415 from Embase, 5576 from Web of Science, 1618 from Scopus, and 3192 from PubMed. After duplicate removal, 6041 records were screened according to titles and abstracts, leaving a sample of 197 papers for full text evaluation (the studies excluded and their exclusion criteria were listed in the Supplementary Information). Finally, 50 primary-level studies meeting all eligibility criteria were included for qualitative evaluation and meta-analysis [18,19,20,21,22,23,24,25,26,27,28,29,30,31,32,33,34,35,36,37,38,39,40,41,42,43,44,45,46,47,48,49,50,51,52,53,54,55,56,57,58,59,60,61,62,63,64,65,66,67].

### 2.2. Study Characteristics

Table 1 summarizes the main characteristics of our study sample, and Table S2 (Supplementary Information) exhibits in detail the variables gathered from primary-level studies. These 50 studies, recruiting a total of 4631 patients (range: 9–429 patients), were published between 1988 and 2022. All studies were observational retrospective cohorts and applied immunohistochemistry in order to assess EGFR overexpression (n = 50, respectively). In relation to the experimental methods, the use of anti-EGFR antibodies was heterogeneous, the most used being Clone 31G7 (n = 6), Clone 2-18C9 (n = 5), D38B1 (n = 4), and Clone H11 (n = 4), with a dominant use of a cut point equal to 10% (n = 14) analyzed in the cell membrane (n = 29). A total of 16 studies processed their antibodies at dilutions <1:100, while 16 >1:100 and were incubated overnight (n = 12) or 1 h (n = 9), at 4 °C (n = 11) or room temperature (n = 9).

### 2.3. Qualitative Evaluation

**Figure 2:**
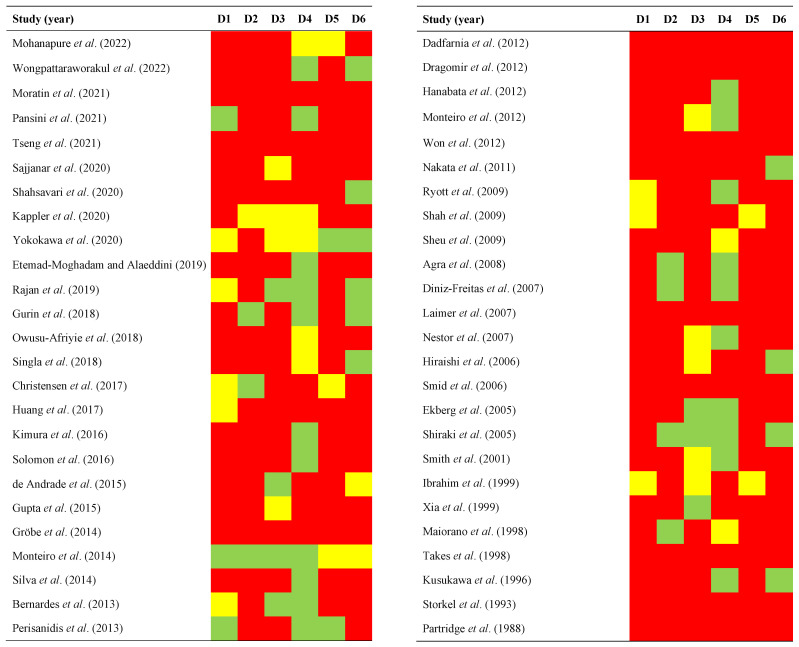
Quality plot graphically representing the risk of bias (RoB) across primary-level studies using a method specifically designed for systematic reviews and meta-analyses addressing questions on prognostic factor studies (i.e., Quality in Prognosis Studies -QUIPS- tool, developed by members of the Cochrane Prognosis Methods Group [68]). The following domains (D1–D6) were critically judged: D1, study participation; D2, study attrition; D3, prognostic factor measurement; D4, outcome measurement; D5, study confounding; and D6, statistical analysis/reporting. RoB was assessed for all domains throughout all studies and scored as potentially low (depicted as green color), moderate (yellow color), or high (red color) [18,19,20,21,22,23,24,25,26,27,28,29,30,31,32,33,34,35,36,37,38,39,40,41,42,43,44,45,46,47,48,49,50,51,52,53,54,55,56,57,58,59,60,61,62,63,64,65,66,67].

The qualitative analysis was conducted using the QUIPS tool (Figure 2), which evaluates potential sources of bias in six domains:

*Study participation*. The risk of this bias was high in 78.00% of the reviewed studies, moderate in 16.00%, and low in 6.00%. Studies often did not report relevant data on the cohorts of oral cancer patients under study (e.g., alcohol and tobacco use, period and place of recruitment, etc.).

*Study attrition*. The risk of this bias was high in 42.00% of the studies, moderate in 2.00%, and low in 14.00%. The lack of reporting of essential data on patients’ follow-up periods was also a common finding (total months, average periods, etc.).

*Prognostic factor measurement*. RoB was high in 44.00% of the studies, moderate in 14.00%, and low in 42.00%. Failure to report data important to the performance and repeatability of experimental methods (e.g., anti-EGFR antibodies, dilution, temperature, or incubation time) was frequent across the studies.

*Outcome measurement*. RoB was high in 14.29% of the studies, moderate in 28.57%, and low in 57.14%. Several studies do not clearly communicate the TNM staging system used, subject to periodic changes, or the clear definition of the survival endpoints under investigation, which are not standardized and currently lack international consensus.

*Study confounding*. RoB was high in 86.00% of the studies, moderate in 10.00%, and low in 4.00%. Relevant potentially confounding variables were frequently not taken into consideration in the study design, such as gender, age, or even relevant clinicopathological variables like histological grade or clinical stage with well-known prognostic value.

*Statistical analysis and reporting*. RoB was high in 76.00% of the studies, moderate in 4.00%, and low in 20.00%. Frequently, survival analyses did not report essential metrics such as hazards ratios with confidence intervals. More serious problems were linked to inappropriate statistical analyses, leading to erroneous results and conclusions.

### 2.4. Quantitative Evaluation (Meta-Analysis)

#### 2.4.1. Association between EGFR Overexpression and Prognostic Variables

**Figure 3:**
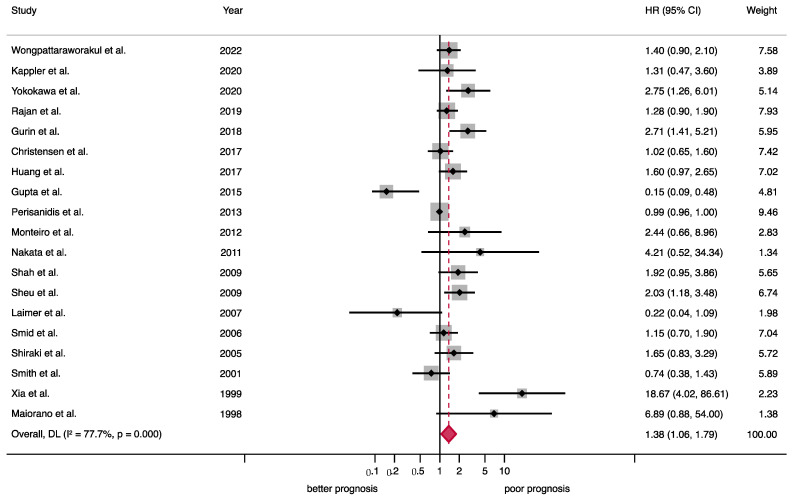
Forest plot graphically representing the meta-analysis on the association between EGFR overexpression and OS in patients with OSCC. Random-effects model, inverse-variance weighting (based on the DerSimonian and Laird method). A HR > 1 suggests that EGFR overexpression is associated with poor prognosis. Diamonds indicate the pooled HRs with their corresponding 95%CIs. Abbreviations: EGFR, epidermal growth factor receptor; OS, overall survival; OSCC, oral squamous cell carcinoma; HR, hazard ratio; CI, confidence intervals [19,23,24,25,28,34,40,42,43,45,48,51,53,55,56,59,65,66,67].

*Overall survival (OS).* EGFR overexpression was significantly associated with a poor survival rate in patients with OSCC (HR = 1.38, 95%CI = 1.06–1.79, *p* = 0.001), and considerable statistical heterogeneity was present (*p* < 0.001, *I*^2^ = 77.7%) (Table 2, Figure 3). This result was derived from a meta-analyzed sample of 19 out of the 50 (38.00%) primary-level studies included in the present systematic review.

*Disease-free survival (DFS).* Close to significant results were found between poor DFS and EGFR overexpression (HR = 1.22, 95%CI = 0.98–1.53, *p* = 0.08), and considerable statistical heterogeneity was also present (*p* < 0.001, *I*^2^ = 65.0%) (Table 2, Figure S15 in Supplementary Information).

#### 2.4.2. Association between EGFR Overexpression and Clinicopathological Variables

Similar significant results were also found for EGFR overexpression with poor differentiated OSCCs (OR = 1.43, 95%CI = 1.05–1.94, *p* = 0.02) and with N+ status (OR = 1.37, 95%CI = 1.01–1.86, *p* = 0.04), only showing moderate heterogeneity was the last parameter (*p* = 0.06, *I*^2^ = 33.6%). On the other hand, EGFR overexpression was not significantly associated with a higher T status (OR = 1.17, 95%CI = 0.72–1.90, *p* = 0.53) or an advanced clinical stage (OR = 1.12, 95%CI = 0.68–1.84, *p* = 0.65) (Table 2, Figures S16–S19, in Supplementary Information).

### 2.5. Quantitative Evaluation (Secondary Analyses)

*Subgroup meta-analysis*. The significant association found between EGFR overexpression and poor OS was also maintained by several subgroups after the stratified meta-analysis (anti-EGFR antibody dilution > 1:100: HR = 1.56, 95%CI = 1.04–2.33, *p* = 0.03; anti–EGFR antibody dilution < 1:100: HR = 1.59, 95%CI = 1.21–2.11, *p* = 0.001; room temperature incubation: HR = 2.93, 95%CI = 1.16–7.38, *p* = 0.02; anti–EGFR antibody Clone 31G7: HR = 2.12, 95% = 1.26–3.59, *p* = 0.005; anti–EGFR antibody Clone H11: HR = 1.33, 95%CI = 1.01–1.76, *p* = 0.05; anti–EGFR antibody D38B1: HR = 2.05, 95%CI = 1.00–4.17, *p* = 0.05; cut–off point of 10%: HR = 1.62, 95%CI = 1.24–2.11, *p* < 0.001, high RoB: HR = 1.63, 95%CI = 1.14–2.31, *p* = 0.007; low RoB: HR = 1.83, 95%CI = 1.12–2.98, *p* = 0.02) (Table 2, Figures S1–S8 in Supplementary Information).

*Meta-regression analysis*. The potential impact of additional study covariates, follow-up period, sex, age, clinical stage, and tobacco and alcohol consumption, on the association between OS and EGFR overexpression was also analyzed, and no significant differences were found (*p* > 0.05 for all covariates) (Table 2, Figures S9–S14 in Supplementary Information).

*Analysis of small-study effects*. Visual inspection analysis of the funnel plots’ asymmetry and the statistical tests conducted for the same purpose confirmed the absence of small-study effects across clinicopathological variables (T status: p_Egger_ = 0.51, N status: p_Egger_ = 0.26; clinical stage: p_Egger_ = 0.90; histological grade: p_Egger_ = 0.98), while significant results were found for prognostic variables, where publication bias could not be ruled out (OS: p_Egger_ = 0.03, DFS: p_Egger_ = 0.08) (Figures S20–S25 in Supplementary Information).

## 3. Discussion

Our systematic review and meta-analysis on the prognostic implications of EGFR overexpression in oral cancer, conducted on 50 studies and 4631 patients, points out that there is an association with lower overall survival (HR = 1.38, 95%CI = 1.06–1.79, *p* = 0.02), higher probability of developing neck lymph node metastases (OR = 1.37, 95%CI = 1.01–1.86, *p* = 0.04), and higher risk of developing poorly differentiated tumors (OR = 1.43, 95% CI = 1.05–1.94, *p* = 0.02). Constitutive oncogenic activation of EGFR is the main mechanism for the acquisition of one of the essential hallmarks of oral cancer, i.e., the ability of tumor cells to maintain a sustained proliferation [2,3,4], which in turn conditions the cells to enter a state of genomic instability that facilitates the acquisition of new additive oncogenic alterations, new hallmarks, which will be clonally transmitted to their progeny. Constitutive activation of EGFR is essentially driven by gene amplification [6,69]; this leads to the formation of dimers between EGFR receptors in the cell membrane and the activation of pro-proliferative intracellular pathways, most notably MAPK and PI3K/Akt, leading to the activation of proliferative genes, essentially but not exclusively *CCND1*, which encodes the proliferation-stimulating protein cyclin D1 [10,11,70]. The oncogenic mechanism linked to the constitutive activation of EGFR is relevant in oral carcinogenesis, as it occurs in other human neoplasms in which, on average, 50–70% of malignant cells overexpress EGFR [6,17,69,71]. In our study, 56.39% of the tumors overexpressed EGFR. The frequency of activation of this oncogenic mechanism in oral carcinogenesis has justified that this protein is one of the few molecules selected as a therapeutic target in this neoplasm (cetuximab) [17]. However, despite its relevance, there is very little evidence-based information, in the form of systematic reviews and meta-analyses, on its prognostic implications [4]. There is only one systematic review and meta-analysis related to the prognostic implications of EGFR2 (ErbB2) overexpression in oral cancer, which reports its association with decreased survival and increased metastatic involvement of the neck lymph nodes [72]. Our meta-analysis, performed on the triple of studies and cases, shows similar results. The influence of EGFR overexpression on survival is obtained when 10% of EGFR+ tumor cells are used as a cut-off point, whereas cut-off points higher than 10% are not discriminative in this sense. This probably indicates that, once an oral carcinoma is established and developed, it is not the hyperproliferative state that is the essential driver of the acquisition of survival-worsening capabilities, but that others, such as the ability to invade and metastasize, resistance to cell death, etc., may then operate independently of the hyperproliferative state. Therefore, if this is so, how could the association found in our study, and in the previous meta-analysis [72], be explained, between EGFR overexpression and N+ status? We believe that this finding may depend on some emerging functions of cyclin D1 associated with invasion and thus with the metastatic capacity of a tumor. Our research group has recently reported [73] that cytoplasmic overexpression of cyclin D1 in oral tumor cells is significantly associated with invasive morphology and the development of actin-based protrusive structures lamellipodia and invadopodia through sequential EGFR-cyclin D1-CDK4/6-paxillin-Rac1 activation, this being an oncogenic pathway that links an essentially proliferative pathway (EGFR) with the increased metastatic capacity of tumor cells.

According to our critical qualitative/risk of bias (RoB) analysis, performed with the QUIPS tool [68] (developed by members of the Cochrane Prognostic Methods Group [74]), the studies presented a very similar design, but not all the same methodological rigor. As is usually found in observational studies, the main RoB source was associated with the lack of control of potentially confounding factors, which were not considered in the design or not integrated in the statistical analyses. Future studies should be better designed, correctly measuring and clearly reporting data related to essential clinical factors that were inconsistently published (e.g., tobacco and alcohol use). Important clinicopathologic or therapeutic variables with prognostic value were also not reported by primary-level studies, such as the number of surgical and non-surgical cases or the presence/absence of distant metastases. These variables sometimes are not published, or typically reported as aggregated data. Therefore, the influence of these covariates could not be quantitatively evaluated through meta-regressions or adjusted through subgroup meta-analyses. Consequently, as another recommendation of our systematic review, future studies should carefully report the clinicodemographical variables of interest, preferably via individual participant data, in order to increase the transparency and scientific quality of the published datasets. It should also be mentioned that in our RoB stratified subgroup meta-analysis, we found the largest effect size between worse survival and a higher methodological quality. This is an important fact that shows that the more carefully designed studies are able to better demonstrate the association between EGFR overexpression and poor prognosis in oral cancer. The methodological recommendations derived from the present systematic review are therefore strongly recommended in order to improve and standardize future research.

Some potential limitations of our systematic review and meta-analysis should also be discussed. First, a considerable statistical heterogeneity degree was observed in the meta-analysis on overall survival. Subsequent stratified meta-analyses revealed more homogeneous subgroups, indicating that potential explanatory sources of heterogeneity are inherent to the variability of experimental methods, singularly differences in anti-EGFR antibodies, cut-off points, and antibody dilutions. Second, the presence of publication bias could not be ruled out for all the variables investigated. Nevertheless, this is a real challenge hard to overcome in the current biomedical research era, where a model of publications of consistently positive results is firmly established [75]. Despite the above limitations, our study was carefully designed and developed following high methodological standards, and presents promising results, being the first meta-analysis to date specifically researching the prognostic implications of EGFR in oral cancer.

## 4. Materials and Methods

This systematic review and meta-analysis followed PRISMA and MOOSE reporting guidelines [76,77], and closely complied with the criteria of Cochrane Prognosis Methods Group [74] and Cochrane Handbook for Systematic Reviews of Interventions [78].

### 4.1. Protocol

To reduce bias risk and improve the transparency, accuracy, and integrity of this study, we previously registered the methodology protocol in the PROSPERO International prospective register of systematic reviews (www.crd.york.ac.uk/PROSPERO, registration number ID433551; accessed on 18 June 2023). The protocol is also consistent with the PRISMA-P Guidelines to ensure a strict approach [79].

### 4.2. Search Strategy

In order to perform the search, MEDLINE/Pubmed, Embase, Web of Science, and Scopus were the main databases employed. Only studies published before the search date (November 2022) were considered. The search was conducted by combining thesaurus terms used in databases (i.e., MeSH and EMTREE) with free terms, designed aiming to increase sensitivity and adapted to the syntax of each database consulted (Table S1 in Supplementary Information). We also manually examined the reference lists of the retrieved studies for additional relevant studies. All references are managed using Mendeley v1.17.10 software (Elsevier, Amsterdam, The Netherlands), and duplicate references were removed.

### 4.3. Eligibility Criteria

The following inclusion criteria were selected: original primary-level studies without restrictions by language, publication date, follow up periods, geographical area, age, or sex; evaluation of EGFR overexpression in OSCC; analysis of the association with at least one of the following prognostics and/or clinicopathological outcomes: overall survival (OS), disease-free survival (DFS), tumor size, N status, clinical stage, or histological grade. OS was defined as the time elapsed from the date of diagnosis/surgery to the date of death by any cause. DFS was defined as the time elapsed from diagnosis/surgery to the detection of locoregional or distant recurrence or to death without recurrence. Given the lack of international consensus standards to define survival endpoints in oncology research, any study using the terms OS/DFS was included, or by using other terms in compliance with our precedent definitions.

Studies meeting at least one of the following criteria were excluded: retracted articles, preclinical research (in vitro research or in vivo animal experimentation), case reports, editorials, letters, meeting abstracts, personal opinions, comments, book chapters, or secondary/tertiary-evidence level studies (systematic reviews, meta-analyses, scoping reviews, umbrella or overviews of reviews, etc.); squamous cell carcinomas from anatomic areas distinct to the oral cavity, and/or tumors of different histopathological lineage; no analysis of the main prognostic or clinicopathological outcomes of interest; lack or insufficient data for the estimation of statistical effect size metrics with their corresponding confidence intervals; and inter-study overlapping populations, determined by verifying the authors’ names and affiliations, source of patients, and recruitment periods. To identify potential overlapping populations, the authors’ names, affiliations, and recruitment period and settings were examined. In cases where the study was conducted by the same research group, we have included the most recent research, or the most complete data published.

### 4.4. Study Selection Process

A team of three blinded authors (JCO, DCG, and VSD) applied the eligibility criteria. A supervising author (PRG) was consulted to resolve any dissimilarities. Article selection was performed in two stages; the first stage consisted of screening the titles and abstracts of retrieved studies in an initial selection, and then reading the full text of selected papers, followed by excluding those that did not meet the inclusion criteria. The reviewers were first jointly trained and calibrated for the process of identifying and selecting studies for several screening rounds, achieving an excellent inter-rater reliability, as measured by calculating a Cohen’s kappa statistic (κ > 0.90).

### 4.5. Data Extraction

After full text reading, authors independently extracted data from the selected articles and used a standardized data collection form using the software Excel (v.16/2018, Microsoft. Redmond, WA, USA). The extracted data sets were secondarily cross-checked together, with discrepancies resolved by consensus. Using the methods proposed by Luo et al. (2018) and Wan et al. (2014) [80,81], data expressed as medians, interquartile range, and/or maximum-minimum values were calculated and converted to means and standard deviation (SD). In cases where it was desirable to combine two or more different datasets expressed as means-standard deviation of subgroups into a single group, the Cochrane Handbook formula was applied [78]. Data were collected on first author, language and publication date, country, sample size, anatomical cancer subsite, sex and age of patients, tobacco use, areca nut and alcohol consumption, recruitment and follow-up period, experimental methods, and relative frequency of EGFR overexpression. Finally, the data necessary to analyze the clinicopathological outcomes was investigated.

### 4.6. Evaluation of Quality and Risk of Bias

The authors used the Quality in Prognosis Studies (QUIPS) tool (developed by members of the Cochrane Prognosis Methods Group [68]) to critically appraise the methodological quality and risk of bias of the studies at the primary level. The following six areas of potential bias were examined: (1) study participation; (2) study attrition; (3) prognostic factor measurement; (4) outcome measurement; (5) study confounding; and (6) statistical analysis/reporting. For each domain, the risk of bias was assessed as low, moderate, or high. Finally, to obtain an overall risk of bias score, an overall score was also estimated based on a method previously described by our research group [82,83,84,85].

### 4.7. Effect Measures

Odds ratios (OR) with their corresponding 95% confidence intervals (CI) were used as an effect measure for clinicopathological outcomes. EGFR overexpression was analyzed as a dichotomous categorical variable according to the scoring systems adopted by primary-level studies. Hazard ratios (HR) with 95%CI were used, due to the time-to-event nature, for the survival outcomes [86]. When authors directly reported HR and 95%CI, these were extracted from primary-level studies. If HR and/or 95%CI were not explicitly provided by the authors, we calculated them using standardized appropriated statistics [86,87]. If these results were only reported through survival curves, datasets were extracted from Kaplan-Meier curves with Engauge Digitizer 4.1 software (open-source digitizing software developed by M. Mitchell).

### 4.8. Synthesis Methods

The primary-level studies included in this systematic review reported different outcomes of interest. Thus, the number of studies and patients was variable for each meta-analysis performed for survival outcomes (overall survival and disease-free survival) and clinicopathological variables (T status, N status, clinical stage, and histological grade). Forest plots were constructed for these outcomes in order to display the results of individual studies, as well as the magnitude, precision, and direction of effects of pooled estimates derived from meta-analytical techniques. All meta-analyses were conducted using the inverse-variance method under a random-effects model (based on the DerSimonian and Laird method). This approach was *a priori* planned in our study protocol in order to account for the possibility that there are different underlying effects among study subpopulations (e.g., differences inherent to the variability of experimental methods, such as different anti-EGFR antibodies, dilutions, or incubation time). All analyses were run in the software Stata v. 16.1 (StataCorp, College Station, TX, USA).

The presence and extent of statistical heterogeneity was assessed using the χ^2^-based Cochran’s Q test. Given the low statistical power of Q-test, *p* < 0.10 was considered significant. We also applied the Higgins *I*^2^ statistic to estimate what proportion of the variance in observed effects reflects the variation in true effects, rather than sampling error. The percentage of inter-study heterogeneity was quantified considering values of 50–75% as a moderate-to-high degree of inconsistency [88,89]. The possible causes of heterogeneity among studies were explored across subgroup meta-analyses and univariable random-effect meta-regression analyses using the restricted maximum likelihood (REML) method [90]. Due to the low number of observations reported by primary-level studies for secondary covariates included in meta-regressions, the *p*-values were re-calculated using a permutation test based on Monte Carlo simulations [91]. To obtain sufficient precision, the number of permutations was 10,000 [92]. Weighted bubble plots were also constructed to graphically represent the fitted meta-regression lines. Finally, in order to assess small-study effects, we planned to generate funnel plots [93] for meta-analyses. Furthermore, the Egger regression test was performed to statistically investigate the asymmetry of funnel plots (performing a linear regression of the effect estimates on their standard errors, weighting by 1/[variance of the effect estimate], considering a p_Egger_-value < 0.10 as significant) [94].

## 5. Conclusions

In conclusion, our meta-analysis provides an evidence-based report that EGFR overexpression is a very frequent oncogenic mechanism in oral oncogenesis that is associated with a worse survival rate and a higher risk of developing lymph node metastases. Our results suggest that immunohistochemical detection of EGFR should be routinely included in the prognostic evaluation of patients with oral cancer, using 10% of EGFR+ tumor cells as a cut-off point to consider a positive case.

## Figures and Tables

**Table 1:** Summarized study characteristics.

Summarized Characteristics of the Study Sample	
Total	50 studies
Year of publication	1988–2022
Total patients (range)	4631 (9—429)
Study design	
Retrospective cohort	50 studies
Experimental methods for EGFR expression determination	
Immunohistochemistry	50 studies
Anti-EGFR antibody	
Clone 31G7	6 studies
Clone 2-18C9	5 studies
Clone H11	4 studies
D38B1	4 studies
Clone 25	2 studies
Clone EP-22	2 studies
sc-03	2 studies
Ab-1	1 study
Ab-4	1 study
Clone 111.6	1 study
Clone 29.1	1 study
Clone 5B7	1 study
Clone SP9	1 study
E30	1 study
HPA018530	1 study
RPN 513	1 study
sc-003	1 study
Not reported	15 studies
Anti-EGFR antibody dilution	
>1:100	16 studies
<1:100	16 studies
Not reported	18 studies
Anti-EGFR antibody incubation time	
Overnight	12 studies
1 h	9 studies
Other	6 studies
Not reported	23 studies
Anti-EGFR antibody incubation temperature	
4 °C	11 studies
Room temperature	9 studies
Not reported	30 studies
Cut-off point	
>10	5 studies
10	14 studies
0	4 studies
Intensity-based	25 studies
Not reported	2 studies
Immunostaining pattern	
Membrane	29 studies
Membrane-cytoplasm	12 studies
Membrane-cytoplasm-nucleus	1 study
Not reported	8 studies
Geographical region	
Asian countries	20 studies
Non-Asian countries	30 studies

**Table 2:** Meta-analyses of prognostic and clinicopathological significance of EGFR overexpression in OSCC.

Meta-Analyses	No. of Studies	No. of Patients	Stat. Model	Wt	Pooled Data		Heterogeneity	
					ES (95% CI)	*p*-Value	*P_het_*	*I*^2^ (%)
**SURVIVAL PARAMETERS**								
**Overall survival**								
EGFR overexpression (all) ^a^	19	2256	REM	D-L	HR = 1.38 (1.06–1.79)	0.02	<0.001	77.7
Subgroup analysis by geographical area ^b^								
Asian	8	1053	REM	D-L	HR = 1.79 (0.89–3.58)	0.10	<0.001	84.2
Non-Asian	11	1203	REM	D-L	HR = 1.20 (0.95–1.52)	0.12	0.01	57.0
Subgroup analysis by anti-EGFR antibody dilution ^b^								
>100	6	743	REM	D-L	HR = 1.56 (1.04–2.33)	0.03	<0.001	80.2
<100	6	689	REM	D-L	HR = 1.59 (1.21–2.11)	0.001	0.60	0.0
Not reported	7	824	REM	D-L	HR = 0.95 (0.44–2.03)	0.89	<0.001	85.3
Subgroup analysis by anti-EGFR antibody incubation time ^b^								
1 h	1	208	—	—	HR = 2.75 (1.26–6.01)	0.01	—	0.0
Overnight	5	616	REM	D-L	HR = 0.86 (0.40–1.83)	0.70	<0.001	84.7
Other	4	506	REM	D-L	HR = 1.96 (0.93–4.10)	0.08	0.004	77.9
Not reported	9	926	REM	D-L	HR = 1.52 (1.04–2.22)	0.03	<0.001	73.8
Subgroup analysis by anti-EGFR antibody incubation temperature^b^								
4 °C	4	451	REM	D-L	HR = 0.79 (0.28–2.23)	0.65	<0.001	88.2
Room temperature	4	523	REM	D-L	HR = 2.93 (1.16–7.38)	0.02	0.004	77.5
Not reported	11	1282	REM	D-L	HR = 1.38 (1.04–1.82)	0.02	0.001	67.6
Subgroup analysis by anti-EGFR antibody ^b^								
Clone 111.6	1	135	—	—	HR = 1.92 (0.95–3.86)	0.07	—	0.0
Clone 2-18C9	1	63	—	—	HR = 2.44 (0.66–8.96)	0.18	—	0.0
Clone 25	1	135	—	—	HR = 1.60 (0.97–2.64)	0.07	—	0.0
Clone 29.1	1	100	—	—	HR = 6.89 (0.88–53.97)	0.07	—	0.0
Clone 31G7	2	204	REM	D-L	HR = 2.12 (1.26–3.59)	0.005	0.51	0.0
Clone 5B7	1	77	—	—	HR = 2.71 (1.41–5.21)	0.003	—	0.0
Clone EP-22	1	120	—	—	HR = 0.15 (0.06–0.35)	<0.001	—	0.0
Clone H11	2	284	REM	D-L	HR = 1.33 (1.01–1.76)	0.05	0.76	0.0
D38B1	2	253	REM	D-L	HR = 2.05 (1.00–4.17)	0.05	0.26	22.0
E30	1	56	—	—	HR = 0.74 (0.38–1.44)	0.37	—	0.0
sc-003	1	111	—	—	HR = 18.67 (4.02–86.66)	<0.001	—	0.0
sc-03	1	140	—	—	HR = 1.65 (0.83–3.29)	0.15	—	0.0
Not reported	4	578	REM	D-L	HR = 0.99 (0–85–1.16)	0.93	0.31	15.5
Subgroup analysis by cut-off point ^b^								
10	7	704	REM	D-L	HR = 1.62 (1.24–2.11)	<0.001	0.32	13.8
>10	3	320	REM	D-L	HR = 2.15 (0.07–61.90)	0.66	<0.001	94.0
Intensity-based	9	1232	REM	D-L	HR = 1.24 (0.95–1.63)	0.12	0.03	65.5
Subgroup analysis by immunostaining pattern ^b^								
Membrane	10	1138	REM	D-L	HR = 1.31 (0.98–1.74)	0.07	0.001	67.0
Mixed membrane-cytoplasm	6	669	REM	D-L	HR = 2.02 (0.61–6.70)	0.25	<0.001	88.2
Not reported	3	449	REM	D-L	HR = 1.39 (0.97–2.01)	0.08	0.16	45.7
Subgroup analysis by overall risk of bias in primary-level studies ^b^								
Low RoB	6	776	REM	D-L	HR = 1.83 (1.12–2.98)	0.02	<0.001	81.0
Moderate RoB	5	547	REM	D-L	HR = 0.79 (0.37–1.67)	0.53	<0.001	82.8
High RoB	8	933	REM	D-L	HR = 1.63 (1.14–2.31)	0.007	0.06	48.5
Univariable meta-regressions by study design and patients characteristics ^c^								
Follow up (months, average)	6	1419	random-effects meta-regression		Coef = 0.000 (−0.032 to 0.032)	0.96 ±0.002 ^d^	het_explained_ = −64.29% ^e^	
Sex (proportion of males, %)	18	2141	random-effects meta-regression		Coef = −0.010 (−0.056 to 0.036)	0.66 ±0.005 ^d^	het_explained_ = −13.93% ^e^	
Age (years, mean)	16	1968	random-effects meta-regression		Coef = −0.003 (−0.108 to 0.102)	0.98 ±0.001 ^d^	het_explained_ = −14.39% ^e^	
Clinical stage (proportion of stage-III/IV patients,%)	7	1032	random-effects meta-regression		Coef = −0.005 (−0.036 to 0.024)	0.56 ±0.005 ^d^	het_explained_ = −225.16% ^e^	
Tobacco consumption (proportion of smokers, %)	9	12707	random-effects meta-regression		Coef = −0.008 (−0.032 to 0.016)	0.48 ±0.005 ^d^	het_explained_ = −13.06% ^e^	
Areca nut/Betel quid consumption (proportion right chewers, %)	2	250	—		—	—	—	
Alcohol consumption (% of patients with positive habit)	5	660	random-effects meta-regression		Coef = −0.047 (−0.111 to 0.018)	0.25 ±0.004 ^d^	het_explained_ = 77.90% ^e^	
**Disease-free survival**								
EGFR overexpression (all) ^a^	19	2320	REM	D-L	HR = 1.22 (0.28–1.53)	0.08	<0.001	65.0
**CLINICOPATHOLOGICAL CHARACTERISTICS**								
**T status**								
EGFR overexpression (all) ^a^	20	1565	REM	D-L	OR = 1.17 (0.72–1.90)	0.53	<0.001	65.0
**N status**								
EGFR overexpression (all) ^a^	24	2040	REM	D-L	OR = 1.37 (1.01–1.86)	0.04	0.06	33.6
**Clinical Stage**								
EGFR overexpression (all) ^a^	18	1456	REM	D-L	OR = 1.12 (0.68–1.84)	0.65	<0.001	65.2
**Histological grade**								
EGFR overexpression (all) ^a^	25	1860	REM	D-L	OR = 1.43 (1.05–1.94)	0.02	0.14	23.8

Abbreviations: Stat., statistical; Wt, method of weighting; ES, effect size estimation; HR, hazard ratio; OR, odds ratio; CI, confidence intervals; REM, random-effects model; D-L, DerSimonian and Laird method; OSCC, oral squamous cell carcinoma; RoB, risk of bias; EGFR, epidermal growth factor receptor. ^a^—Meta-analysis of aggregate (summary) data. ^b^—Subgroup meta-analysis. ^c^—Meta-regression analysis of the potential effect of study covariates on the association between EGFR overexpression and overall survival in OSCC. A meta-regression coefficient >0 indicates a greater impact of covariates on poor prognosis. ^d^—*p*-value ± standard error recalculated after 10,000 permutations based on Montecarlo simulations. ^e^—Proportion of between-study variance explained (adjusted R^2^ statistic) using the residual maximum likelihood (REML) method. A negative number for the proportion of heterogeneity explained reflects no heterogeneity explained.

## Data Availability

Data is contained within the article or supplementary material .

## Supplementary Materials

The following supporting information can be downloaded at: https://www.mdpi.com/article/10.3390/ijms241511888/s1.
