# Supplementary material for: Prognostic and Clinicopathological Significance of Epidermal Growth Factor Receptor (EGFR) Expression in Oral Squamous Cell Carcinoma: Systematic Review and Meta-Analysis
Source: Int J Mol Sci. 2023 Jul 25;24(15):11888. doi: 10.3390/ijms241511888 (PMC10419199; doi:10.3390/ijms241511888)
Supplement: Supplementary file 1 [file ijms-24-11888-s001.zip › ijms-2491306-supplementary.pdf]

Supplementary information to the manuscript

Prognostic and Clinicopathological Significance of Epidermal Growth Factor Receptor (EGFR) Expression in Oral Squamous Cell Carcinoma: Systematic Review and Meta-Analysis

Table of contents

1. Search strategy. Table S1. 3
2. Characteristics of analyzed studies. Table S2 4
3. Meta-analysis on the of EGFR overexpression and overall survival in OSCC............8
    3.1 Subgroup meta-analysis by geographical area...8
    3.2 Subgroup meta-analysis by anti-EGFR antibody dilution 9
    3.3 Subgroup meta-analysis by anti-EGFR antibody incubation time..................10
    3.4 Subgroup meta-analysis by anti-EGFR antibody incubation temperature.......11
    3.5 Subgroup meta-analysis by antibody 12
    3.6 Subgroup meta-analysis by cut-off point 13
    3.7 Subgroup meta-analysis by immunohistochemical staining pattern................14
    3.8 Subgroup meta-analysis by overall risk of bias in primary-level studies 15
    3.9 Univariable meta-regression on the effect of follow up 16
    3.10 Univariable meta-regression on the effect of sex..17
    3.11 Univariable meta-regression on the effect of age 18
    3.12 Univariable meta-regression on the effect of stage.......................................19
    3.13 Univariable meta-regression on the effect of tobacco...................................20
    3.14 Univariable meta-regression on the effect of alcohol 21
4. Meta-analysis of EGFR overexpression and DFS in OSCC 22
5. Meta-analysis of EGFR overexpression and T status...23
6. Meta-analysis of EGFR overexpression and N status 24
7. Meta-analysis of EGFR overexpression and clinical stage 25
8. Meta-analysis of EGFR overexpression and histological grade.................................26
9. Analysis of small-study effects. 27
    9.1 EGFR overexpression and overall survival in OSCC 27
    9.2 EGFR overexpression and DFS in OSCC..28
    9.3 EGFR overexpression and T status in OSCC 29
    9.4 EGFR overexpression and N status in OSCC...30
    9.5 EGFR overexpression and clinical stage in OSCC...31
    9.6 EGFR overexpression and histological grade in OSCC 32
10. List of excluded studies with reasons...33

1. Search strategy

Table S1. Search strategy for each database, number of results, and execution date.

Database	Query/Search Strategy	Results/ Items found	Search time limits
PubMed	("Genes, erbB-1"[Mesh] OR "epidermal growth factor receptor"[all fields] OR "EGFR"[all fields] OR "erbb1"[all fields] OR "cerbb1"[all fields] OR "her1"[all fields] OR "pEGFR"[all fields] OR "phospho-EGFR"[all fields]) AND ("mouth"[MeSH] OR "mouth"[All Fields] OR "oral"[All Fields]) AND ("carcinoma, squamous cell"[MeSH] OR ("carcinoma"[All Fields] AND "squamous"[All Fields] AND "cell"[All Fields]) OR "squamous cell carcinoma"[All Fields] OR "Neoplasms"[Mesh] OR neopla*[All Fields] OR "cancer"[All Fields])	3192	November, 2022
Embase	('epidermal growth factor receptor'/exp OR 'epidermal growth factor receptor' OR 'EGFR' OR 'erbb1' OR 'cerbb1' OR 'her1' OR 'pEGFR' OR 'phospho-EGFR') AND ('mouth'/exp OR 'mouth') AND ('squamous cell carcinoma'/exp OR 'carcinoma' OR 'malignant neoplasm'/exp OR 'neoplas*' OR 'cancer')	2415	November, 2022
Web of Science	TS=("epidermal growth factor receptor" OR "EGFR" OR “erbb1” OR "cerbb1" OR "her1" OR "pEGFR" OR "phopho-EGFR") AND TS=(“mouth”) AND TS=(“squamous cell carcinoma” OR neoplas* or “cancer”)	5576	November, 2022
Scopus	TITLE-ABS-KEY("epidermal growth factor receptor" OR "EGFR" OR “erbb1” OR "cerbb1" OR "her1" OR "pEGFR" OR "phopho-EGFR") AND (“mouth” OR “oral”) AND (“squamous cell carcinoma” OR neoplas* or “cancer”))	1618	November, 2022
Total			12801

2. Table S2. Characteristics of analyzed studies (n = 50).

Study	Year	Country	Publ. language	Study design	Recruitment period	Follow up m, mean±SD (range)	Sample Size, n	Sex M, n (%) F, n (%)	Age, mean±SD (range)	Tumour subsites	Tobacco, n (%)	Alcohol, n (%)	Areca, n (%)	Methods	Anti-EGFR antibody (clonality)	Dilution, incubation time, temperature, antigen retrieval	IHQ pattern	IHQ cutoff point (%)	EGFR + (%)
Mohanapure et al.	2022	India	English	Retrospective	2015-2019	NR	70	M: 47 (67.14)<br>F: 23 (32.86)	52.86<br>(35-75)	Buccal mucosa<br>Gingiva<br>Hard palate<br>Lip<br>Retromolar trigone<br>Tongue	41<br>(58.57)	21<br>(30)	7<br>(10)	IHQ	Clone EP-22<br>(monoclonal)	NR	Membrane	10	65<br>(92.86)
Wongpattarawora kul et al.	2022	United States	English	Retrospective	2005-2014	107 ± 37	143<br>Missing=<br>3	M: 85 (58.22)<br>F: 61 (41.78)	NR	Floor of mouth<br>Gingiva<br>Tongue<br>Other	64<br>(44.14)	NR	NR	IHQ	Clone H11<br>(monoclonal)	1:200<br>NR<br>NR	NR	10	59<br>(41.26)
Moratin et al.	2021	Germany	English	Retrospective	2010-2016	NR	222	M: 137 (61.7)<br>F: 85 (38.3)	64.3 ± 11.1<br>(27-88)	Buccal mucosa<br>Floor of mouth<br>Lip<br>Mandible<br>Maxilla<br>Oropharynx<br>Tongue	NR	NR	NR	IHQ	D38B1<br>(monoclonal)	NR	Cytoplasm	33	NR
Pansini et al.	2021	Brazil	English	Retrospective	2010-2017	NR	97	M: 85 (72.0)<br>F: 24 (20.3)	60	Buccal mucosa<br>Floor of mouth<br>Gingiva<br>Hard palate<br>Retromolar trigone<br>Tongue<br>Vestibule of mouth	51<br>(43.2)	52<br>(44.1)	NR	IHQ	D38B1<br>(monoclonal)	1:100<br>2-5 min<br>Room temperature	Membrane<br>Cytoplasm	Labelling index (intensity x cell count)	50<br>(51.55)
Tseng et al.	2021	Taiwan	English	Retrospective	1993-2006	60	429	NR	NR	NR	NR	NR	NR	IHQ	NR	1:50<br>Overnight<br>4ºC	Membrane	NR	105<br>(24.48)
Sajjanar et al.	2020	India	English	Retrospective	NR	NR	34	M: 23 67.65)<br>F: 11 (32.35)	46.8<br>(23-65)	Buccal mucosa<br>Gingiva<br>Tongue<br>Other	NR	NR	NR	IHQ	HPA018530<br>(polyclonal)	1:100<br>60 min<br>NR	Membrane	Labelling index (intensity x cell count)	21<br>(61.76)
Shahsavari et al.	2020	Iran	English	Retrospective	NR	NR	46	M:25 (54.3)<br>F:21 (45.7)	58.63±10.6<br>5	NR	NR	NR	NR	IHQ	NR	1:10<br>60 min<br>NR	Membrane	10	17<br>(73.91)
Kappler et al.	2020	Germany	English	Retrospective	1998-2002	NR	45	M:31 (68.89)<br>F:14 (31.11)	57	NR	NR	NR	NR	IHQ	D38B1<br>(monoclonal)	1:50<br>NR<br>NR	Membrane	10	9<br>(20)
Yokokawa et al.	2020	Japan	English	Retrospective	NR	60	208	M:129 (62)<br>F.79 (38)	59.1	Buccal mucosa<br>Floor of mouth<br>Gingiva	110<br>(52.9)<br>Missing=<br>10	141<br>(67.8)<br>Missing=<br>11	NR	IHQ	D38B1<br>(monoclonal)	NR<br>60 min<br>Room temperature	Membrane	Labelling index (Intensity)	60<br>(28.85)
Etemad-Moghadam and Alaeddini	2019	Iran	English	Retrospective	NR	NR	50	M:28 (58.33)<br>F:20 (41.67)<br>Missing=2	63.3	NR	NR	NR	NR	IHQ	Clone H11<br>(monoclonal)	1:50<br>60 min<br>NR	Membrane	Labelling index (intensity-cell count)	28<br>(56)

Rajan et al.	2019	United States	English	Retrospective	2005-2014	NR (NR- 157)	141	M: 82 (58.16) F: 59 (41.84)	62 (19-81)	Floor of mouth Gingiva Tongue Other	92	NR	NR	IHQ	Clone H11 (monoclonal)	1:200 30 min Room temperature	Membrane	10	71 (50.35)
Gurin et al.	2018	Czech Republic	English	Retrospective	2007-2012	62.7 (54.9-69.9)	77	M: 65 (84.4) F: 12 (15.6)	58.3 (39.6-72.3)	Oropharynx Tongue Other	NR	NR	NR	IHQ	Clone 5B7 (monoclonal)	NR	Membrane	10	37 (48.05)
Owusu-Afriyie et al.	2018	Ghan	English	Retrospective	2006-2014	NR	28	M:19 (67.86) F: 9 (32.14)	55.07 ± 17.33	NR	NR	NR	NR	IHQ	NR	NR	NR	Labelling index (intensity-cell count)	17 (60.71)
Singla et al.	2018	India	English	Retrospective	NR	NR	40	NR	NR	NR	NR	NR	NR	NR	NR	NR	Membrane	10	39 (97.5)
Christensen et al.	2017	Denmark	English	Retrospective	2000-2012	61.2 (1.2-190.8)	191	M: 126 (66) F: 65 (34)	Median 59 (23-89)	Floor of mouth Tongue	159 (83)	NR	NR	IHQ	NR	NR 32 min NR	NR	Labelling index (intensity-cell count)	77 (40.31)
Huang et al.	2017	Taiwan	English	Retrospective	1997 - 2004	96	135 Missing= 59	M:194 (100) F: 0 (0)	49.28 ± 11.34	NR	175 (90.2)	132	175	IHQ	Clone 25 (monoclonal)	1:100 NR NR	Membrane Cytoplasm Nuclear	Labelling index	48 (35.56)
Kimura et al.	2016	Japan	English	Retrospective	1998-2008	NR	24	NR	64.4 ± 11.2 (43-89)	NR	NR	NR	NR	IHQ	Clone 2-18C9 (monoclonal)	NR	Membrane Cytoplasm	10	14 (58.33)
Solomon et al.	2016	India	English	Retrospective	2008-2013	(18-60)	178	M:141 (79.21) F: 37 (20.79)	54.51 ± 13.43 (24-80)	NR	NR	NR	NR	IHQ	Clone 2-18C9 (monoclonal)	1:40 Overnight 4ºC	Membrane	Labelling index (intensity + cell count)	150 (84.27)
de Andrade et al.	2015	Brazil	English	Retrospective	NR	NR	45	NR	NR	Buccal mucosa Gingiva Oropharynx Palate Retromolar trigone Tongue	NR	NR	NR	IHQ	Clone SP9 (monoclonal)	1:200 60 min Room Temperature	Membrane	51	36 (80)
Gupta et al.	2015	India	English	Retrospective	2009-2011	NR (NR-24)	120	M: 96 (80) F: 24 (20)	49.48 ± 12.19 (20-67)	Buccal mucosa Gingiva Hard palate Lip Retromolar trigone Tongue	NR	NR	NR	IHQ	Clone EP-22 (monoclonal)	NR Overnight 4ºC	Membrane Cytoplasm	50	87 (72.5)
Gröbe et al.	2014	Germany	English	Retrospective	1998-2008	NR (NR-120)	206	M: 150 (72.82) F: 56 (27.18)	56 (20-93)	Buccal mucosa Floor of mouth Tongue Maxilla	NR	NR	NR	IHQ	Clone 2-18C9 (monoclonal)	0 NR NR	Membrane Cytoplasm Nuclear	Labelling index (intensity x cell count)	196 (95.15)
Monteiro et al.	2014	Portugal	English	Retrospective	2000-2006	39.6 ± 33.1 (1-142)	74 Missing= 1	M:55 (74.32) F:19 (25.68)	62.3 ± 15.6 (25-96)	Buccal mucosa Floor of mouth Gingiva Hard palate Lip Retromolar trigone Tongue	NR	NR	NR	IHQ	Clone 25 (monoclonal)	1:100 60min Room Temperature	Membrane	Labelling index (intensity x cell count)	68 (93.15)
Silva et al.	2014	Brazil	English	Retrospective	NR	120	82	M:70 (85.37) F:12 (14.63)	54.8 (38-78)	NR	67 (92.1)	63 (87.5)	NR	IHQ	NR	1:400 Overnight 4ºC	Membrane	Labelling index (intensity x cell count)	38 (50)

Bernardes et al.	2013	Brazil	English	Retrospective	NR	6	52	M:12 (23.08)<br>F: 40 (76.92)	56.3	Bucal mucosa<br>Floor of mouth<br>Gingiva<br>Palate<br>Tongue	24	27	NR	IHQ	Clone 31G7<br>(monoclonal)	1:100<br>60 min<br>Room temperature	Membrane	10%	29<br>(55.77)
Perisanidis et al.	2013	Austria	English	Retrospective	2000-2009	55.2	113	M:83 (73.45)<br>F:30 (26.55)	Median<br>58<br>(24-79)	Oral cavity<br>Oropharynx	94	83	NR	IHQ	NR	NR	Membrane	Labelling index (cell count x cell count)	112<br>(99.12)
Dadfarnia et al.	2012	United States	English	Retrospective	1992-2009	NR	16	M:11 (68.75)<br>F:5 (31.25)	56.2<br>(40-73)	Floor of mouth<br>Palate<br>Retromolar trigone Tongue<br>Tonsil	NR	NR	NR	IHQ	Clone H11<br>(monoclonal)	1:200<br>NR<br>NR	Membrane	10%	3<br>(18.75)
Dragomir et al.	2012	Romania	English	Retrospective	2008-2011	NR	44	NR	NR	Lip<br>Palate<br>Tongue	NR	NR	NR	IHQ	NR	NR	Membrane<br>Cytoplasm	0	33<br>(75)
Hanabata et al.	2012	Japan	English	Retrospective	NR	NR	52	M:35 (67.31)<br>F:17 (32.69)	56.75<br>(23-84)	Tongue	NR	NR	NR	IHQ	sc-03<br>(polyclonal)	1:100<br>NR<br>NR	NR	Labelling index (intensity x cell count)	46<br>(88.46)
Monteiro et al.	2012	Spain	English	Retrospective	1995-2003	NR	63<br>Missing=<br>4	M:52 (77.6)<br>F: 15 (22.4)	59 ± 12.6<br>(31-85)	Buccal mucosa<br>Floor of mouth<br>Gingiva<br>Hard palate<br>Retromolar trigone Tongue	NR	NR	NR	IHQ	Clone 2-18C9<br>(monoclonal)	NR<br>30 min<br>Room temperature	Membrane<br>Cytoplasm	10	50<br>(79.37)
Won et al.	2012	South Korea	English	Retrospective	1995-2009	NR	60	M: 38 (63.3)<br>F: 22 (36.7)	56.3 ± 12.1	Buccal mucosa<br>Floor of mouth<br>Palate<br>Oropharynx<br>Tongue<br>Tonsil	36<br>(60)	NR	NR	IHQ	NR	NR	Membrane	Labelling index (intensity x cell count)	46<br>(76.67)
Nakata et al.	2011	Japan	English	Retrospective	1999-2009	NR<br>(NR-128.5)	89	M:62 (69.66)<br>F:27 (30.34)	NR	Tongue	40	29	NR	IHQ	Clone 31G7<br>(monoclonal)	1:50<br>NR<br>NR	Membrane	50	73<br>(82.02)
Ryott et al.	2009	Sweeden	English	Retrospective	200-2004	36	78	M:45 (57.69)<br>F:33 (42.31)	NR	Tongue	40	NR	NR	IHQ	Clone 31G7<br>(monoclonal)	NR	Membrane	Labelling index (intensity x cell count)	74<br>(94.87)
Shah et al.	2009	India	English	Retrospective	2000-2003	NR<br>(24-NR)	135	M:101<br>(74.81)<br>F:34 (25.19)	45<br>(28-75)	Buccal mucosa<br>Tongue	110	NR	NR	IHQ	Clone 111.6<br>(monoclonal)	1:50<br>Overnight<br>4ºC	Membrane<br>Cytoplasm	10	81<br>(60)
Sheu et al.	2009	China	English	Retrospective	NR	60	115	NR	50.27 ±<br>11.38<br>(30-78)	Buccal mucosa<br>Floor of mouth<br>Gingiva<br>Palate<br>Tongue	106<br>(92.2)	69<br>(60)	101<br>(87.8)	IHQ	Clone 31G7<br>(monoclonal)	1:10<br>NR<br>NR	NR	Labelling index (intensity)	29<br>(25.22)
Agra et al.	2008	Brazil	English	Retrospective	1990-2004	Median<br>11.94<br>(0.8-140.9)	111<br>Missing=<br>6	M:81 (72.97)<br>F:30 (27.03)	Median<br>56<br>(31-90)	Buccal mucosa<br>Floor of mouth<br>Gingiva<br>Lip<br>Palate<br>Retromolar trigone Tongue<br>Tonsil	NR	NR	NR	IHQ	NR	NR	Membrane	0	82<br>(77.36)
Diniz-Freitas	2007	Spain	English	Retrospective	1995-2000	Median<br>37.80<br>(6-108)	47	M:39 (82.98)<br>F:8 (17.02)	57.68<br>(31-85)	Buccal mucosa<br>Floor of mouth<br>Hard palate<br>Retromolar trigone Tongue	NR	NR	NR	IHQ	Clone 2-18C9<br>(monoclonal)	NR<br>30 min<br>NR	Membrane	Labelling index (intensity x cell count)	35<br>(74.47)

Laimer et al.	2007	Austria	English	Retrospective	1980-1997	NR (NR-88)	109	M: 83 (76.15) F: 26 (23.85)	Median 63.2 (25.6–86.8)	NR	NR	NR	NR	IHQ	NR	NR	Membrane	Labelling index (intensity x cell count)	100 (91.74)
Nestor et al.	2007	Sweeden	English	Retrospective	NR	NR	9	NR	NR	Buccal mucosa Gingiva Tongue	NR	NR	NR	IHQ	Clone 31G7 (monoclonal)	1:40 NR 4ºC	Membrane	Labelling index (intensity x cell count)	9 (100)
Hiraishi et al.	2006	Japan	English	Retrospective	1990-2002	NR	52	M:33 (63.46) F:19 (36.54)	67.5 (49-91)	Floor of mouth Gingiva Hard palate Maxillary sinus Oropharynx Tongue	NR	NR	NR	IHQ	NR	1:200 Overnight 4ºC	NR	>0%	48 (92.31)
Smid et al.	2006	The Netherlands	English	Retrospective	1985-2000	NR (24-60)	165	M: 109 (66.06) F: 56 (33.94)	Median 58 (25–89)	NR	NR	NR	NR	IHQ	NR	1:20 Overnight NR	Membrane	Labelling index intensity	132 (80)
Ekberg et al.	2005	Sweeden	English	Retrospective	!996-2002	NR	19	M:8 (42.1) F:11 (57.9)	65.4	Floor of mouth Gingiva Palate Tongue	NR	NR	NR	IHQ	Clone 31G7 (monoclonal)	1 :30 960 min 4ºC	Membrane	Labelling index (cell count x intensity)	18 (94.74)
Shiraki et al.	2005	Japan	English	Retrospective	1986-1998	Median 66 (5-134)	140	M:98 (70) F:42 (30)	59 (26-85)	Buccal mucosa Floor of mouth Gingiva Tongue	NR	NR	NR	IHQ	sc-03 (polyclonal)	1:40 Overnight 4ºC	Membrane Cytoplasm	Labelling index (cell count x intensity)	54 (38.57)
Smith et al.	2001	United States	English	Retrospective	1981-1992	73	56	M:46 (82.14) F:10 (17.86)	NR	Oral cavity Oropharynx	NR	NR	NR	IHQ	E30	NR Overnight 4ºC	Membrane	Labelling index (index)	36 (64.29)
Ibrahim et al.	1999	Norway	English	Retrospective	1991-1993	NR	26 Missing= 1	M:18 (69.23) F:8 (30.77)	65.5± 12.83 (24-82)	Floor of mouth. Gingiva. hard palate. tongue	18	10	NR	IHQ	RPN 513	1:20 60 min NR	Membrane Cytoplasm	10	23 (92)
Xia et al.	1999	China	English	Retrospective	1989-1991	NR (NR-74)	111	M:74 (66.6) F: 37 (33.3)	Median 51 (13-76)	Floor of mouth Gingiva Tongue Others	NR	NR	NR	IHQ	sc-003 (polyclonal)	1:200 180 min Room Temperature	Membrane Cytoplasm	20	41 (36.94)
Maiorano et al.	1998	Italy	English	Retrospective	NR	46.8 (5-140)	100	M:79 (79) F:21 (21)	63.4 (28-93)	Buccal mucosa Floor of mouth Gingiva Lip Palate Retromolar trigone Tongue	NR	NR	NR	IHQ	Clone 29.1	1:400 NR NR	Membrane Cytoplasm	10	36
Takes et al.	1998	The Netherlands	English	Retrospective	1990-1995	NR	35 Missing= 1	M:22 (61.1) F:14 (38.9)	60 (34-87)	NR	NR	NR	NR	IHQ	Ab-4	1:20 NR NR	Membrane	>0	18 (51.43)
Kusukawa et al.	1996	Japan	English	Retrospective	NR	NR	65	M:40 (61.54) F:25 (38.46)	60.2 ± 15.2 (27-89)	Floor of mouth Gingiva Lip Mandible Tongue	NR	NR	NR	IHQ	Ab-1	1:400 720 min 4ºC	Cytoplasm	NR	20 (30.77)
Storkel et al.	1993	Germany	English	Retrospective	NR	60	100	NR	NR	NR	NR	NR	NR	IHQ	NR	1:50 NR NR	Membrane	Labelling index (cell count x intensity)	74 (74)
Partridge et al.	1988	United Kingdom	English	Retrospective	NR	NR	20	NR	NR	NR	NR	NR	NR	IHQ	NR	NR 60 min NR	NR	Labelling index	18 (90)

Abbreviations: n, number; m, months; y, years; SD, standard deviation; IHQ, immunohistochemical; NR, not reported; OSCC, oral squamous cell carcinoma.

3. Meta-analysis on the EGFR overexpression and overall survival in OSCC

3.1 Subgroup meta-analysis by geographical area

Figure S1. Forest plot graphically representing the stratified analysis by geographical area on the association between EGFR overexpression and overall survival in patients with OSCC.

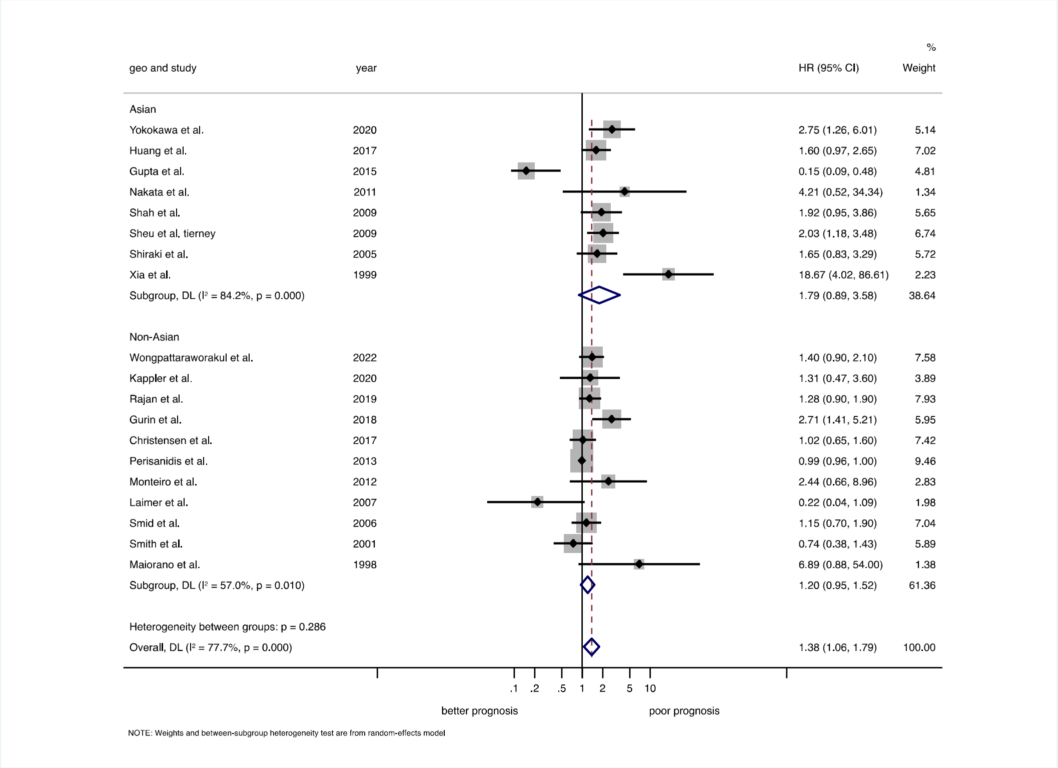

OSCC, oral squamous cell carcinoma; HR, hazard ratio; CI, confidence intervals. Random-effects model, inverse-variance weighting (based on the DerSimonian and Laird method). A HR > 1 suggests that the EGFR overexpression is associated with poor overall survival. Diamonds indicate the pooled HRs with their corresponding 95% CIs.

3.2 Subgroup meta-analysis by anti-EGFR antibody dilution

Figure S2. Forest plot graphically representing the stratified analysis by anti-EGFR antibody dilution on the association between EGFR overexpression and overall survival in patients with OSCC.

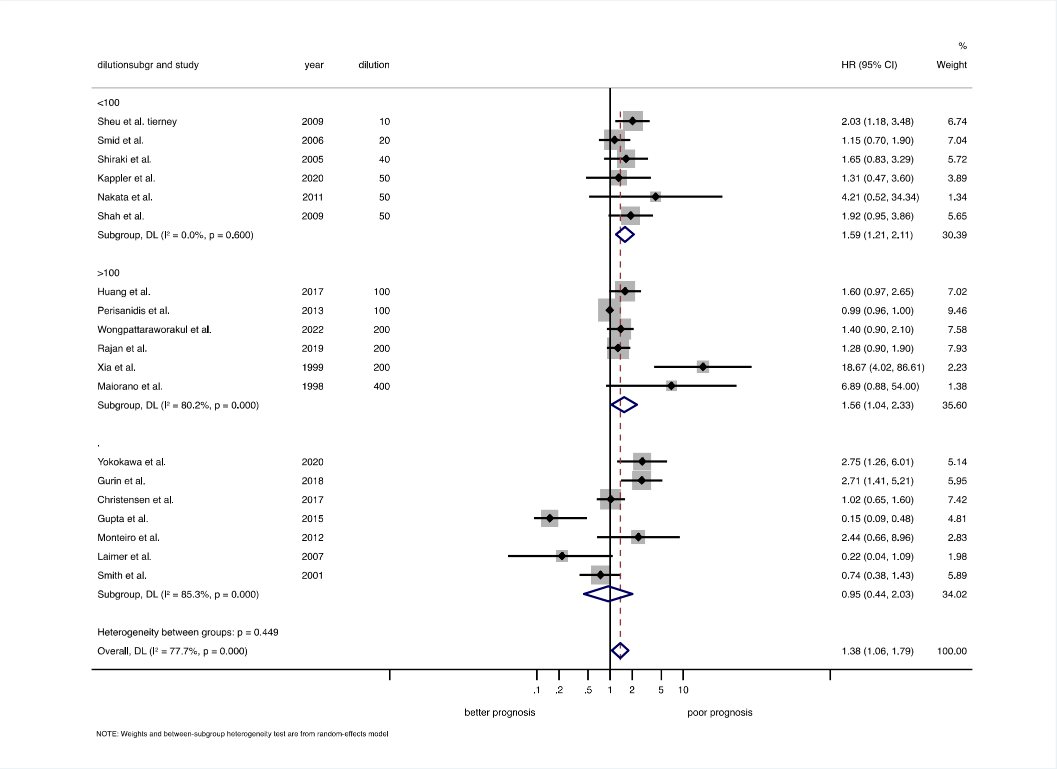

OSCC, oral squamous cell carcinoma; HR, hazard ratio; CI, confidence intervals. Random-effects model, inverse-variance weighting (based on the DerSimonian and Laird method). A HR > 1 suggests that EGFR overexpression is associated with poor overall survival. Diamonds indicate the pooled HRs with their corresponding 95% CIs.

3.3 Subgroup meta-analysis by anti-EGFR antibody incubation time

Figure S3. Forest plot graphically representing the stratified analysis by anti-EGFR antibody incubation time on the association between EGFR overexpression and overall survival in patients with OSCC.

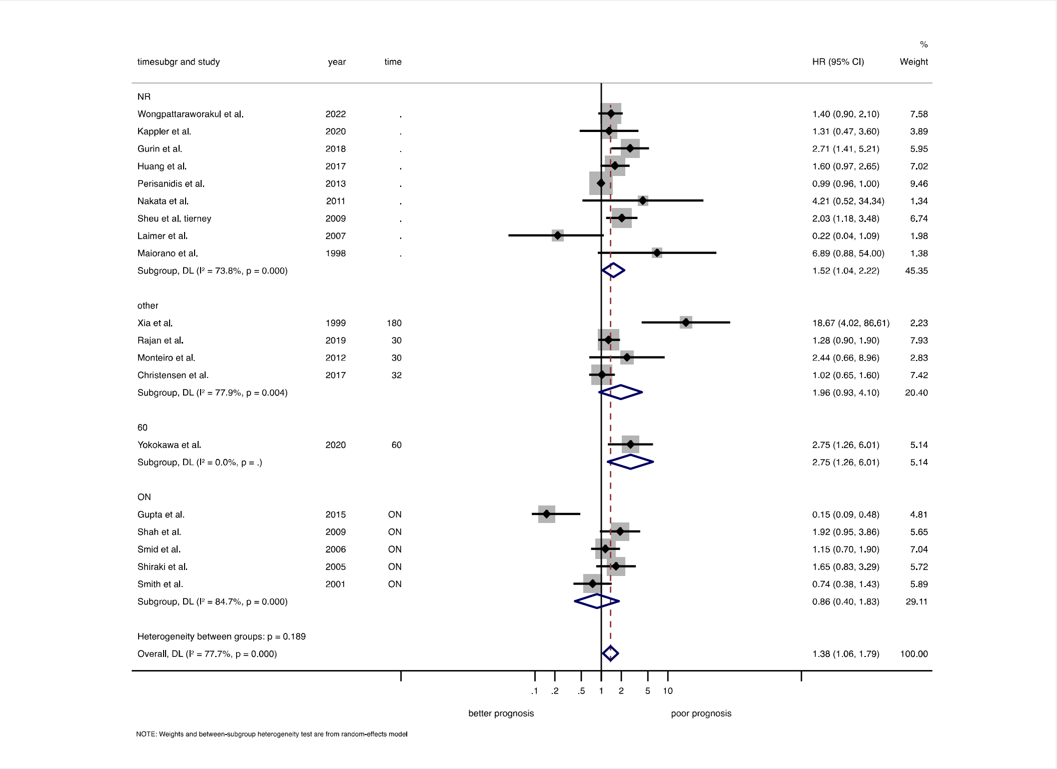

OSCC, oral squamous cell carcinoma; HR, hazard ratio; CI, confidence intervals. Random-effects model, inverse-variance weighting (based on the DerSimonian and Laird method). A HR > 1 suggests that EGFR overexpression is associated with poor overall survival. Diamonds indicate the pooled HRs with their corresponding 95% CIs.

3.4 Subgroup meta-analysis by anti-EGFR antibody incubation temperature

Figure S4. Forest plot graphically representing the stratified analysis by anti-EGFR antibody incubation temperature on the association between EGFR overexpression and overall survival in patients with OSCC.

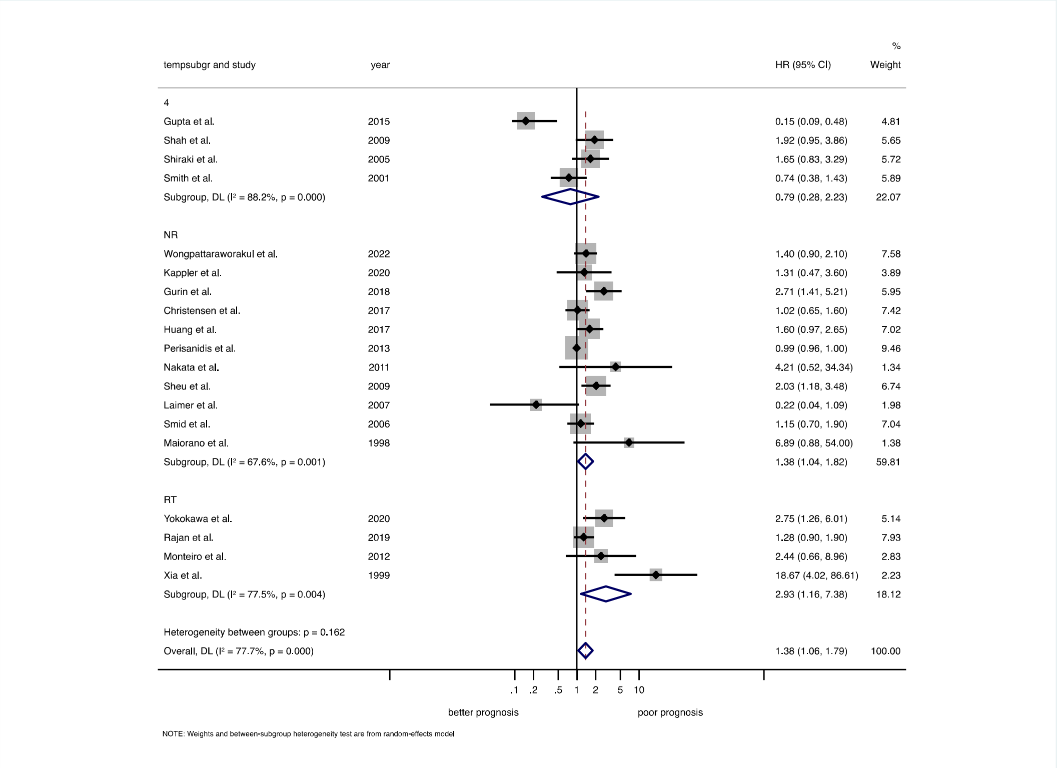

OSCC, oral squamous cell carcinoma; HR, hazard ratio; CI, confidence intervals. Random-effects model, inverse-variance weighting (based on the DerSimonian and Laird method). A HR > 1 suggests that EGFR overexpression is associated with poor overall survival. Diamonds indicate the pooled HRs with their corresponding 95% CIs.

3.5 Subgroup meta-analysis by antibody

Figure S5. Forest plot graphically representing the stratified analysis by antibody on the association EGFR overexpression and overall survival in patients with OSCC.

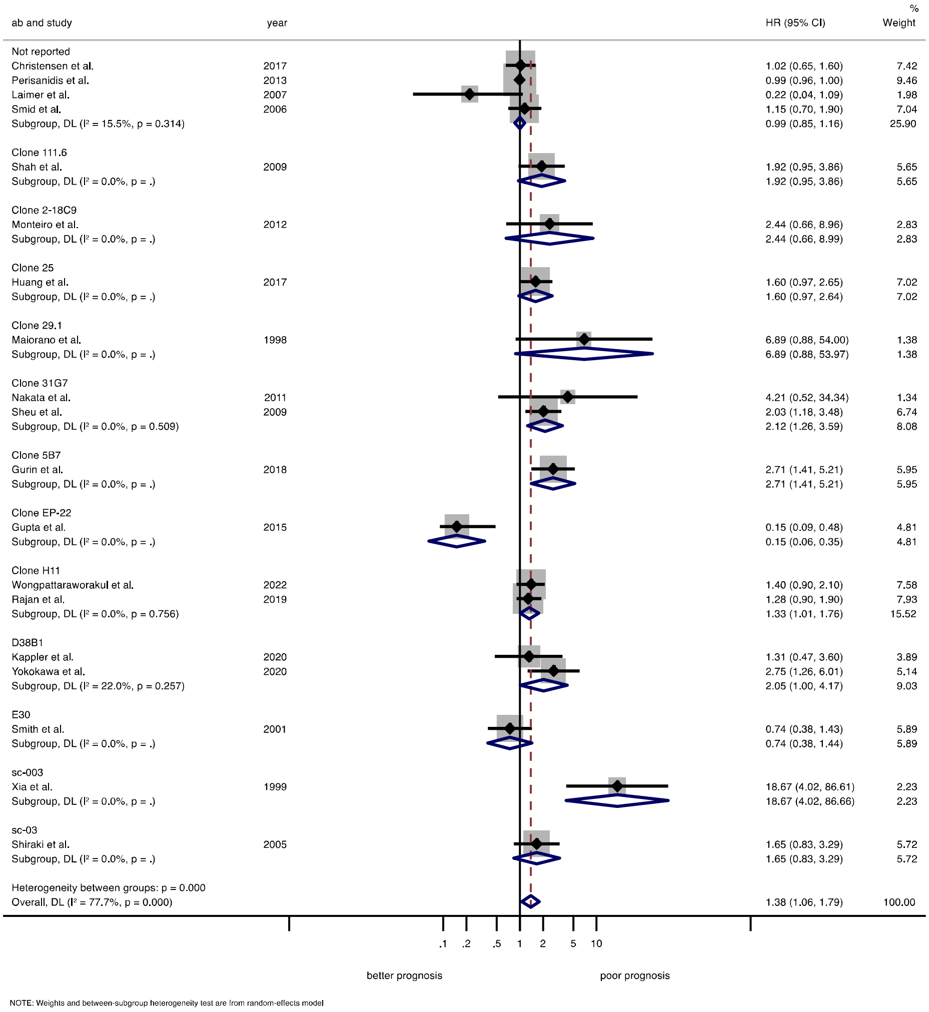

OSCC, oral squamous cell carcinoma; HR, hazard ratio; CI, confidence intervals. Random-effects model, inverse-variance weighting (based on the DerSimonian and Laird method). A HR > 1 suggests EGFR overexpression is associated with poor overall survival. Diamonds indicate the pooled HRs with their corresponding 95% CIs.

3.6 Subgroup meta-analysis by cut-off point

Figure S6. Forest plot graphically representing the stratified analysis by cut-off point on the association EGFR overexpression and overall survival in patients with OSCC.

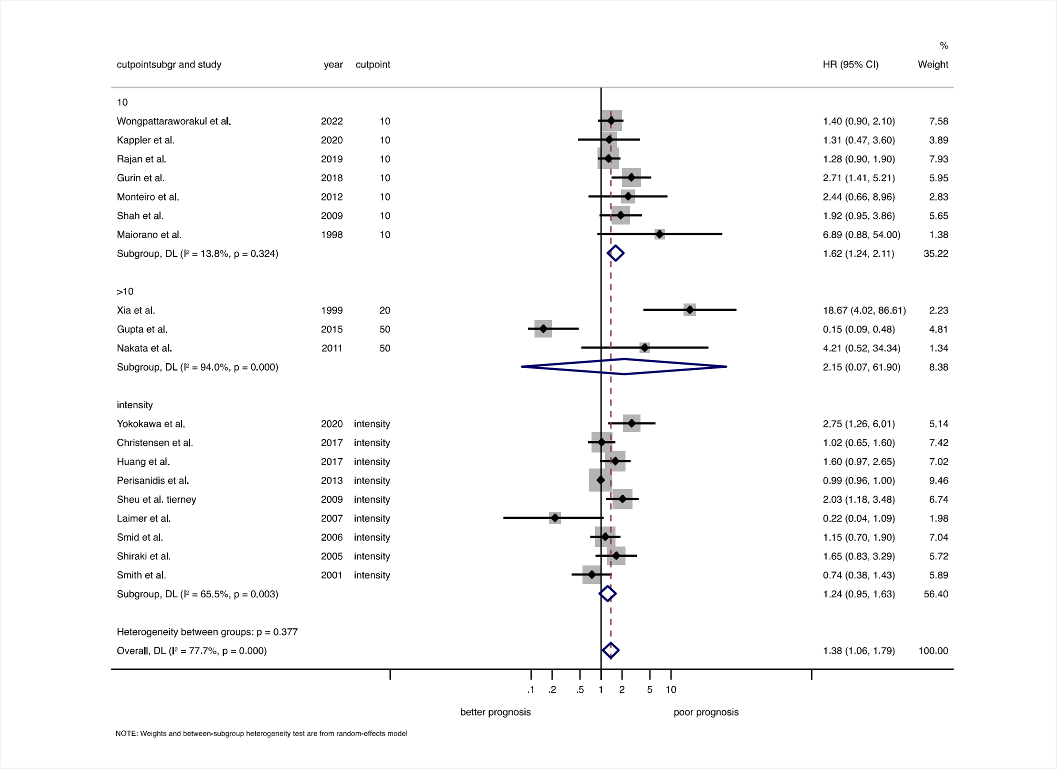

OSCC, oral squamous cell carcinoma; HR, hazard ratio; CI, confidence intervals. Random-effects model, inverse-variance weighting (based on the DerSimonian and Laird method). A HR > 1 suggests EGFR overexpression is associated with poor overall survival. Diamonds indicate the pooled HRs with their corresponding 95% CIs.

3.7 Subgroup meta-analysis by immunohistochemical staining pattern

Figure S7. Forest plot graphically representing the stratified analysis by immunohistochemical staining pattern on the association between EGFR overexpression and overall survival in patient with OSCC.

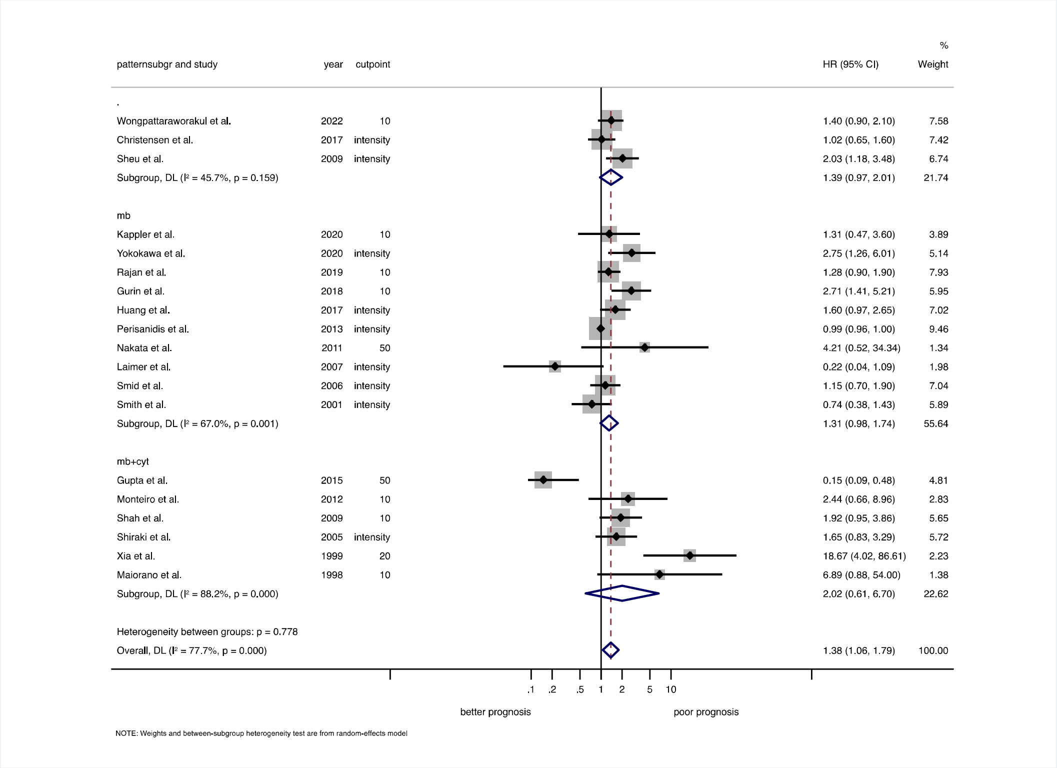

OSCC, oral squamous cell carcinoma; HR, hazard ratio; CI, confidence intervals; mb, membrane; mb+cyt, membrane and cytoplasm. Random-effects model, inverse-variance weighting (based on the DerSimonian and Laird method). A HR > 1 suggests that EGFR overexpression is associated with poor overall survival. Diamonds indicate the pooled HRs with their corresponding 95% CIs.

3.8 Subgroup meta-analysis by overall risk of bias in primary-level studies

Figure S8. Forest plot graphically representing the stratified analysis by overall EGFR in primary-level studies, on the association between EGFR overexpression and overall survival in patients with OSCC.

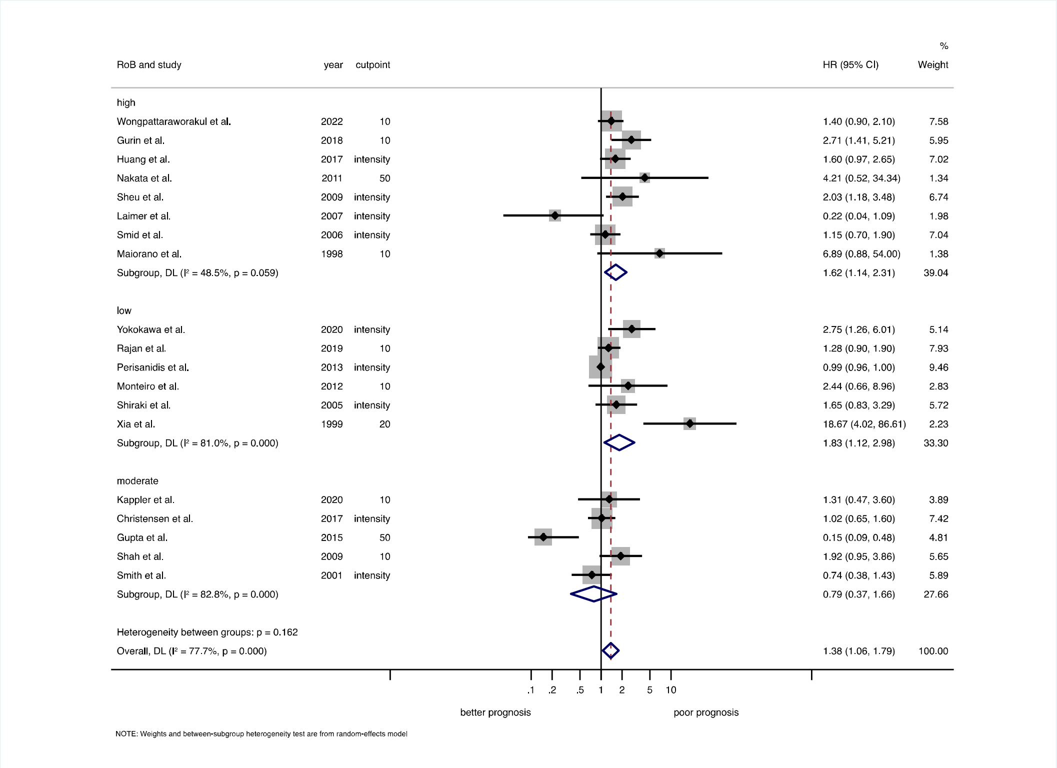

OSCC, oral squamous cell carcinoma; RoB, risk of bias; HR, hazard ratio; CI, confidence intervals. Random-effects model, inverse-variance weighting (based on the DerSimonian and Laird method). A HR > 1 suggests that EGFR overexpression is associated with poor overall survival. Diamonds indicate the pooled HRs with their corresponding 95% CIs.

3.9 Univariable meta-regression on the effect of follow up

Figure S9. Bubble plot graphically representing the univariable meta-regression analysis of the potential effect of follow up period (expressed in months, in x-axis) on the association between EGFR overexpression and overall survival in patients with OSCC (using HR as effect size measure, in y-axis).

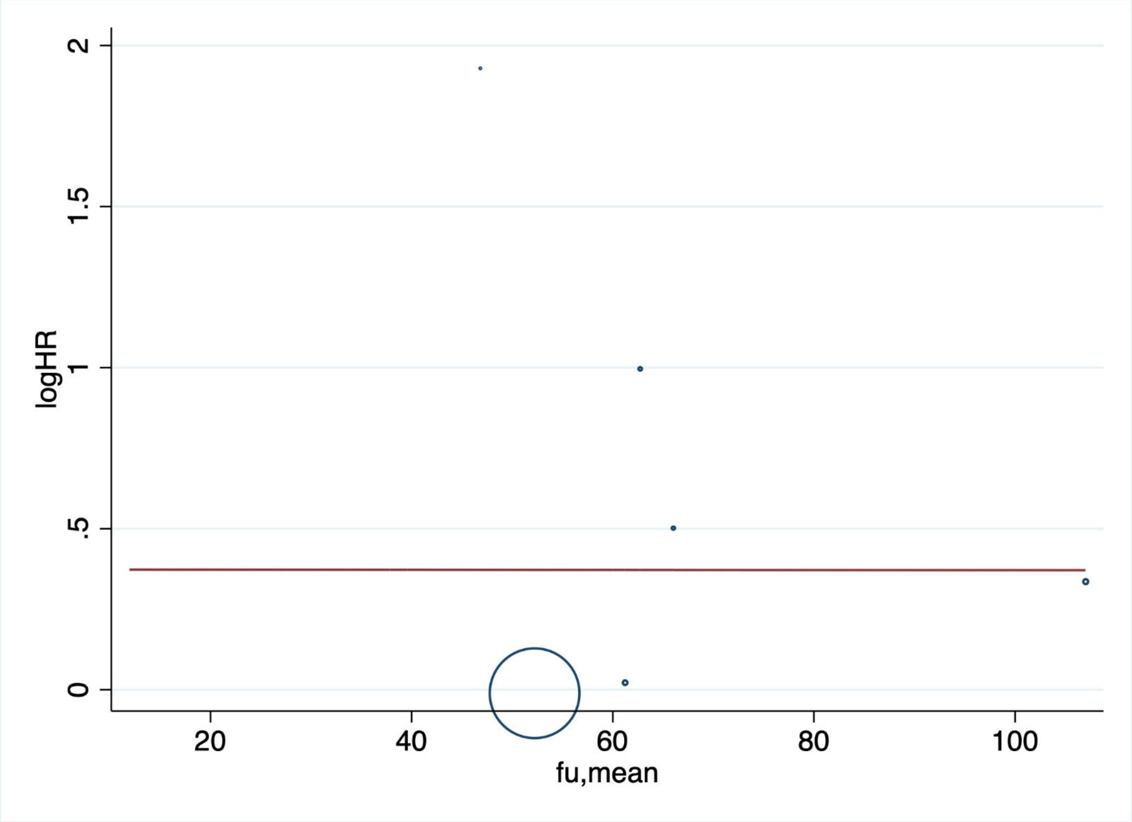

OSCC, oral squamous cell carcinoma; HR, hazard ratio; log, natural logarithm (i.e., log base e). The red line exhibits the fitted regression line together with blue circles representing the estimates from each individual study, sized according to the precision of each estimate (the inverse of its within-study variance).

3.10 Univariable meta-regression on the effect of sex

Figure S10. Bubble plot graphically representing the univariable meta-regression analysis of the potential effect of sex (% of males) on the association EGFR overexpression and overall survival in patients with OSCC.

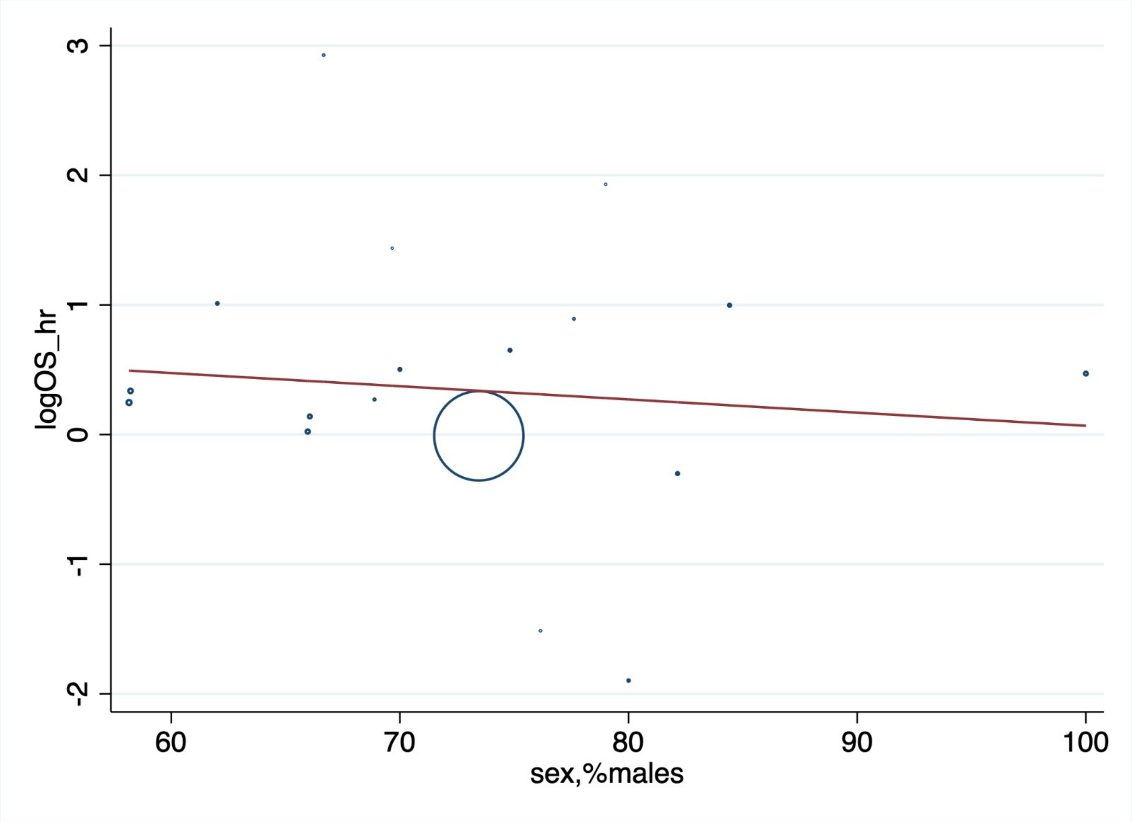

OSCC, oral squamous cell carcinoma; HR, hazard ratio; log, natural logarithm (i.e., log base e). The red line exhibits the fitted regression line together with blue circles representing the estimates from each individual study, sized according to the precision of each estimate (the inverse of its within-study variance).

3.11 Univariable meta-regression on the effect of age

Figure S11. Bubble plot graphically representing the univariable meta-regression analysis of the potential effect of age (mean age of patients, expressed in years) on the association between EGFR overexpression and overall survival in patients with OSCC.

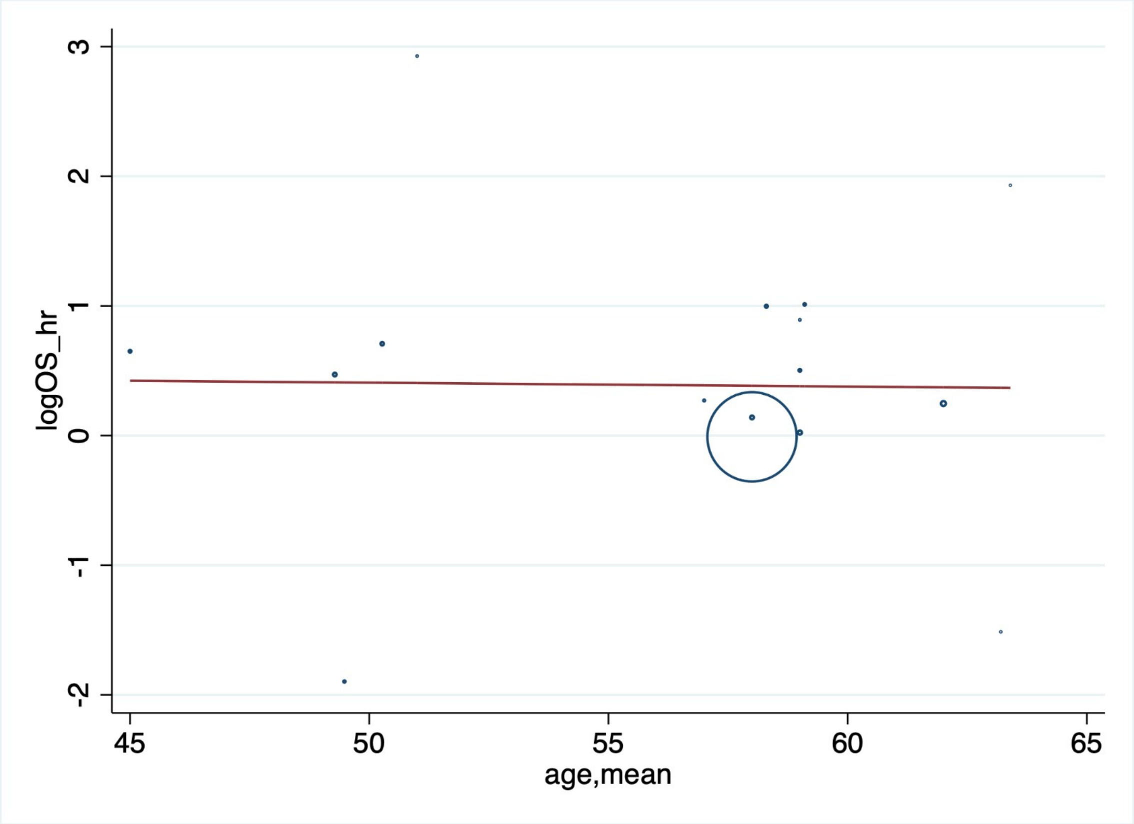

OSCC, oral squamous cell carcinoma; HR, hazard ratio; log, natural logarithm (i.e., log base e). The red line exhibits the fitted regression line together with blue circles representing the estimates from each individual study, sized according to the precision of each estimate (the inverse of its within-study variance).

3.12 Univariable meta-regression on the effect of stage

Figure S12. Bubble plot graphically representing the univariable meta-regression analysis of the potential effect of stage (expressed in percentage, in x-axis) on the association between EGFR overexpression and overall survival in patients with OSCC.

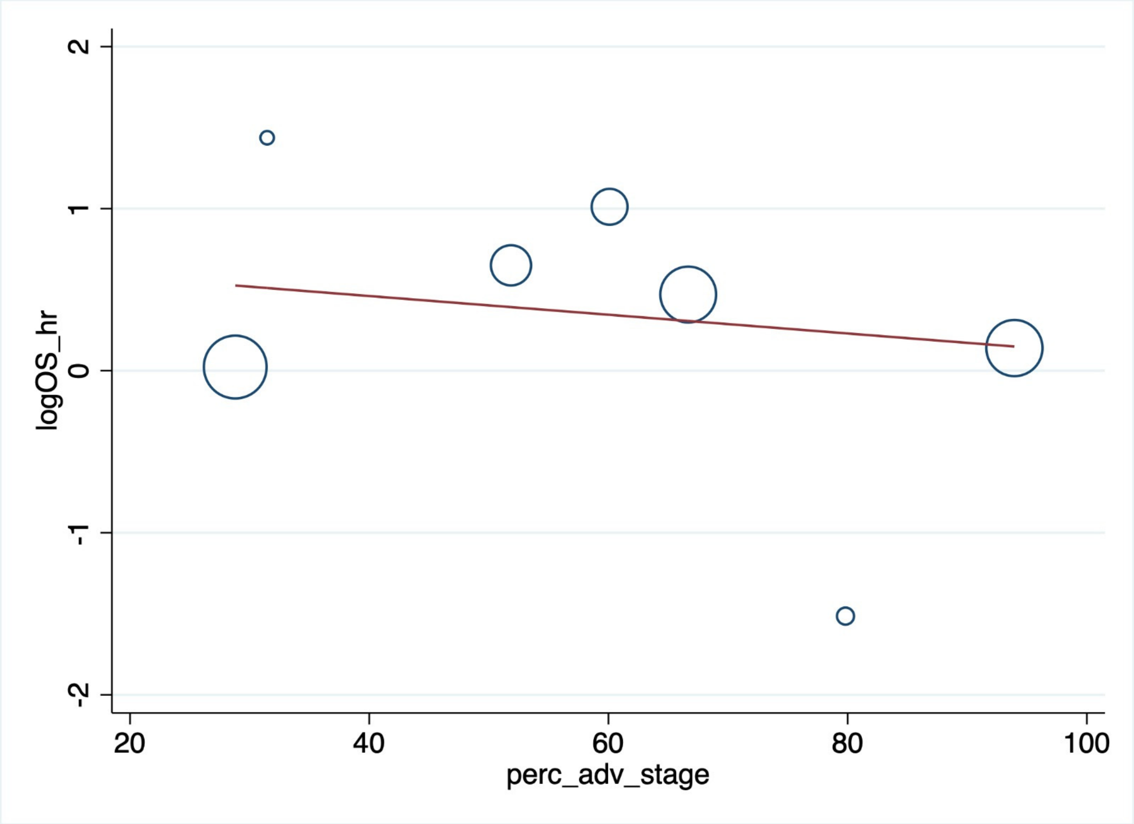

OSCC, oral squamous cell carcinoma; HR, hazard ratio; log, natural logarithm (i.e., log base e). The red line exhibits the fitted regression line together with blue circles representing the estimates from each individual study, sized according to the precision of each estimate (the inverse of its within-study variance).

3.13 Univariable meta-regression on the effect of tobacco

Figure S13. Bubble plot graphically representing the univariable meta-regression analysis of the potential effect of tobacco (percentage of smokers, expressed in x-axis) on the association between EGFR overexpression and overall survival in patients with OSCC.

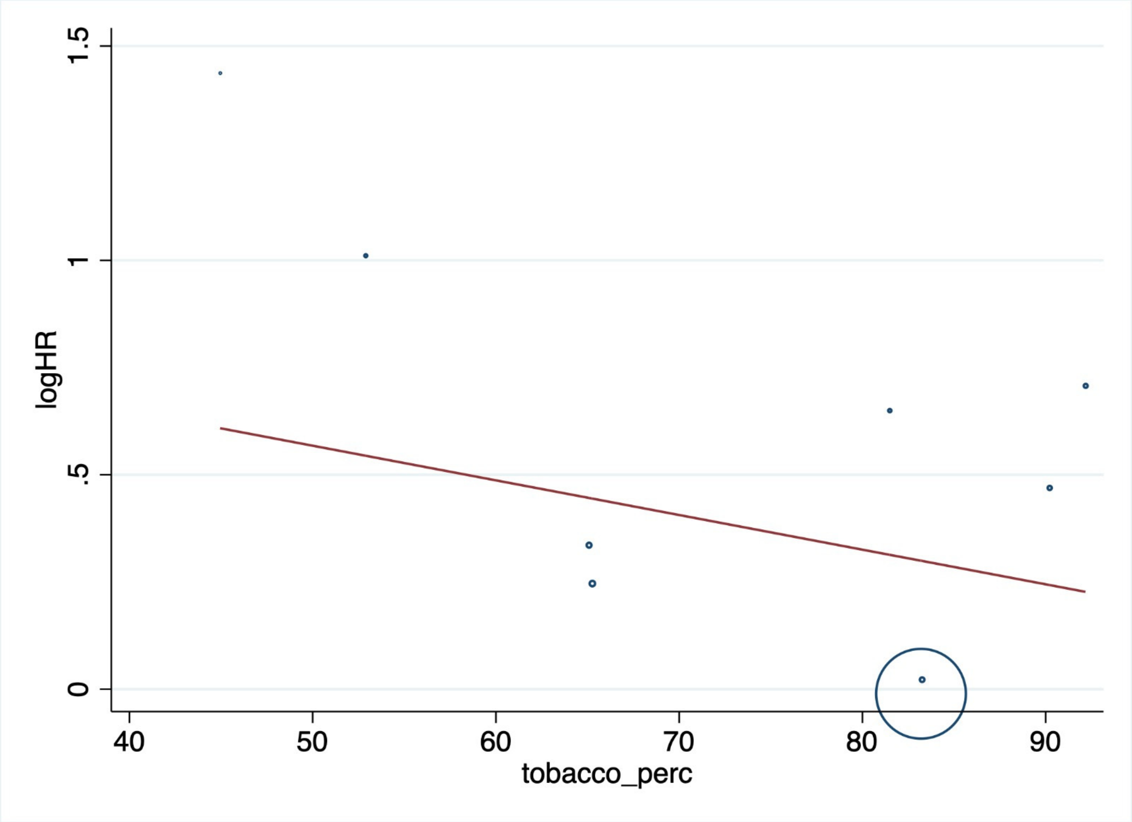

OSCC, oral squamous cell carcinoma; HR, hazard ratio; log, natural logarithm (i.e., log base e). The red line exhibits the fitted regression line together with blue circles representing the estimates from each individual study, sized according to the precision of each estimate (the inverse of its within-study variance).

3.14 Univariable meta-regression on the effect of alcohol

Figure S14. Bubble plot graphically representing the univariable meta-regression analysis of the potential effect of alcohol (percentage of drinkers, expressed in x-axis) on the association between EGFR overexpression and overall survival in patients with OSCC.

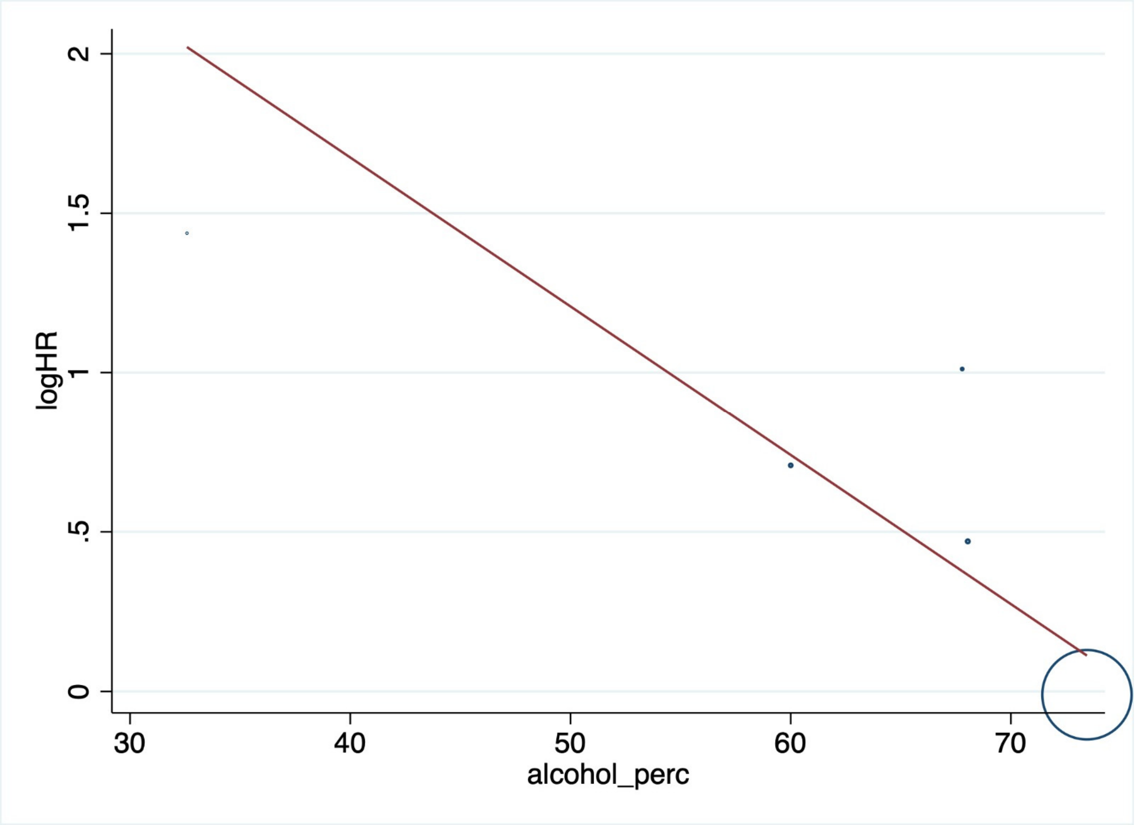

OSCC, oral squamous cell carcinoma; HR, hazard ratio; log, natural logarithm (i.e., log base e). The red line exhibits the fitted regression line together with blue circles representing the estimates from each individual study, sized according to the precision of each estimate (the inverse of its within-study variance).

4. Meta-analysis on EGFR overexpression and DFS in OSCC

Figure S15. Forest plot graphically representing the meta-analysis on the association between EGFR overexpression and DFS in patients with OSCC.

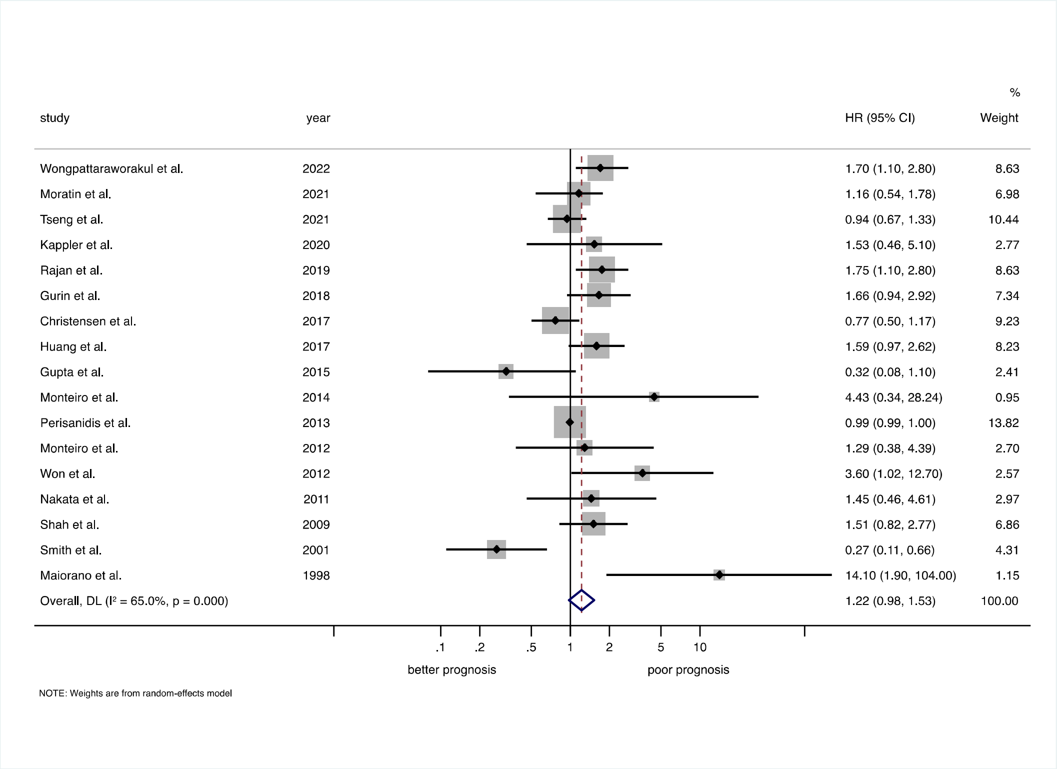

DFS, disease-free survival; OSCC, oral squamous cell carcinoma; HR, hazard ratio; CI, confidence intervals. Random-effects model, inverse-variance weighting (based on the DerSimonian and Laird method). A HR > 1 suggests that EGFR overexpression is associated with poor DFS. Diamonds indicate the pooled HRs with their corresponding 95% CIs.

5. Meta-analysis on EGFR overexpression and T status in OSCC

Figure S16. Forest plot graphically representing the meta-analysis on the association between EGFR overexpression and T status (T3/T4 vs. T1/T2) in patients with OSCC.

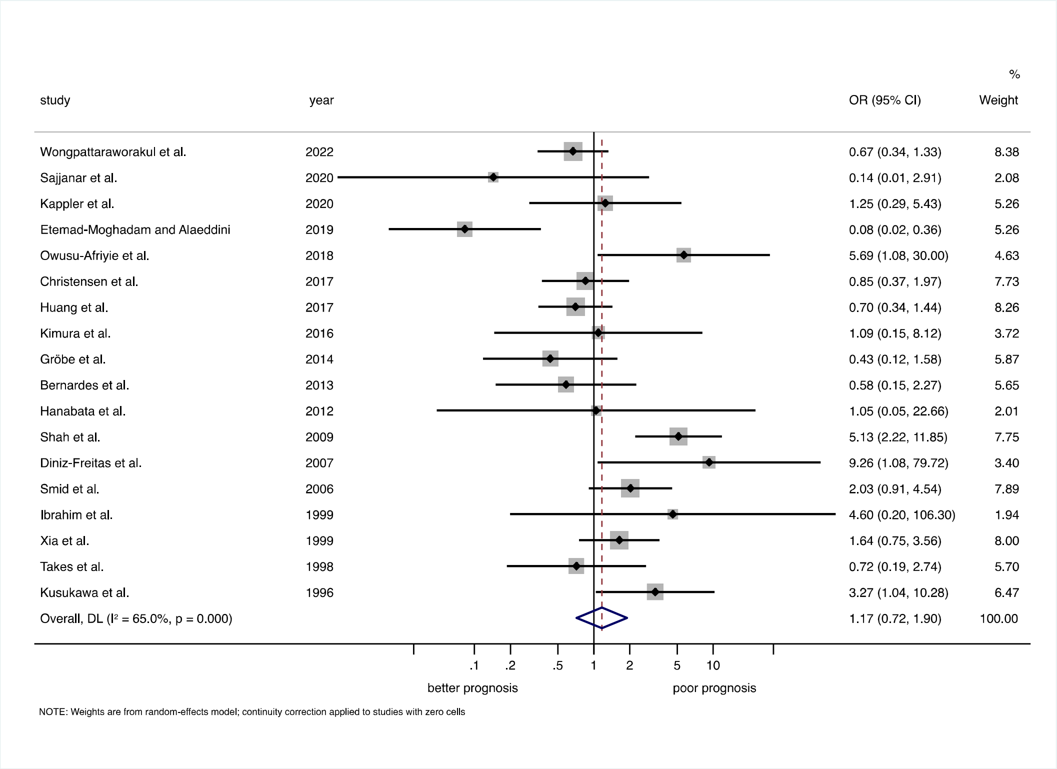

OSCC, oral squamous cell carcinoma; OR, odds ratio; CI, confidence intervals. Random-effects model, inverse-variance weighting (based on the DerSimonian and Laird method). A OR > 1 suggests EGFR overexpression is associated with a higher T status. Diamonds indicate the pooled ORs with their corresponding 95% CIs.

6. Meta-analysis on EGFR overexpression and N status in OSCC

Figure S17. Forest plot graphically representing the meta-analysis on the association between EGFR overexpression and N status (positive metastatic lymph nodes vs. negative) in patients with OSCC.

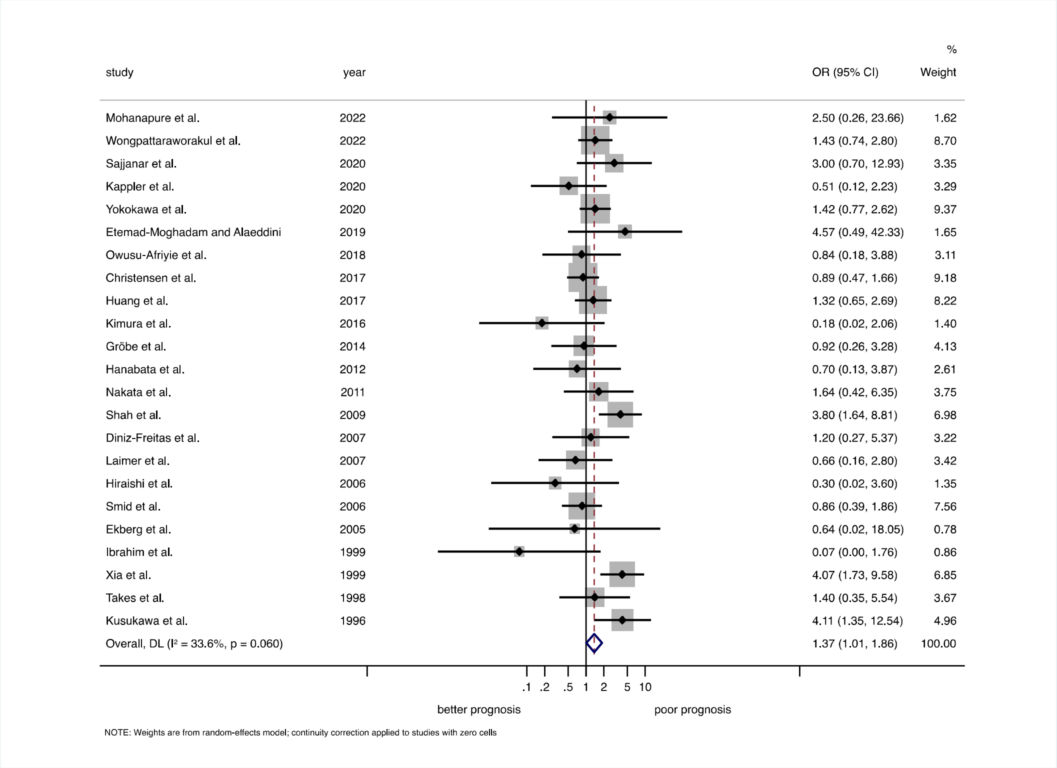

OSCC, oral squamous cell carcinoma; OR, odds ratio; CI, confidence intervals. Random-effects model, inverse-variance weighting (based on the DerSimonian and Laird method). A OR > 1 suggests that EGFR overexpression is associated with positive N status. Diamonds indicate the pooled ORs with their corresponding 95% CIs.

7. Meta-analysis on EGFR overexpression and clinical stage in OSCC

Figure S18. Forest plot graphically representing the meta-analysis on the association between EGFR expression and clinical stage (III/IV vs. I/II) in patients with OSCC.

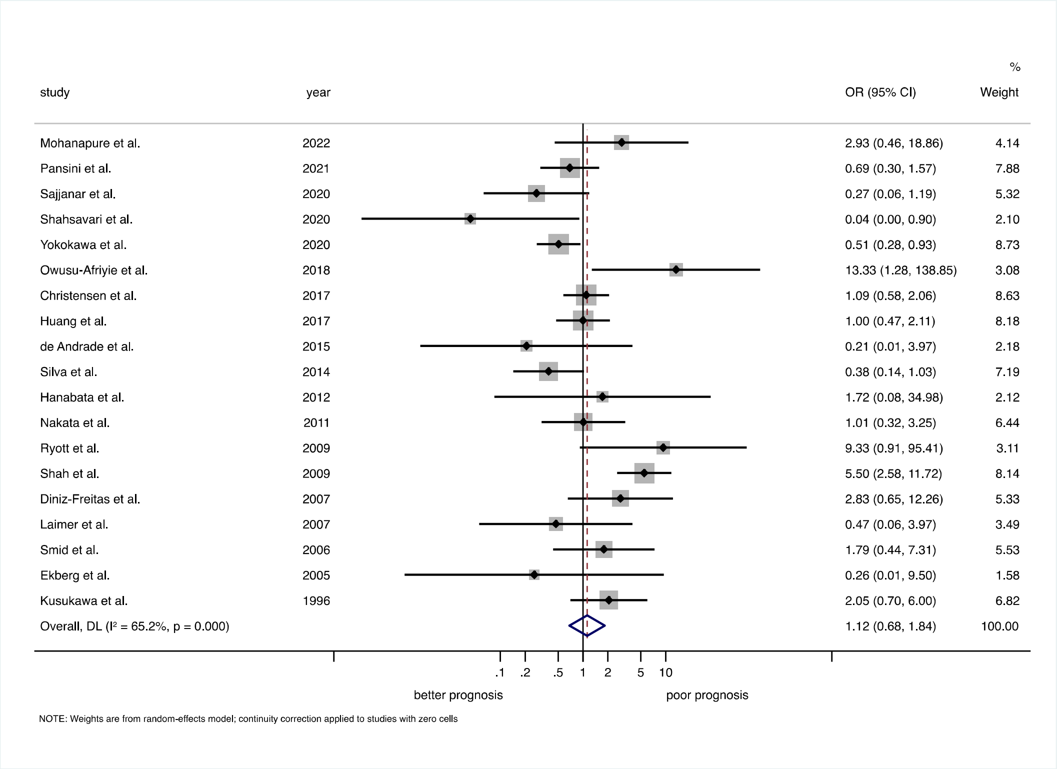

OSCC, oral squamous cell carcinoma; OR, odds ratio; CI, confidence intervals. Random-effects model, inverse-variance weighting (based on the DerSimonian and Laird method). A OR > 1 suggests that EGFR overexpression is associated with a higher stage. Diamonds indicate the pooled ORs with their corresponding 95% CIs.

8. Meta-analysis on EGFR overexpression and histological grade in OSCC

Figure S19. Forest plot graphically representing the meta-analysis on the association between EGFR overexpression and histological grade (poorly-moderate vs. well-differentiated carcinomas) in patients with OSCC.

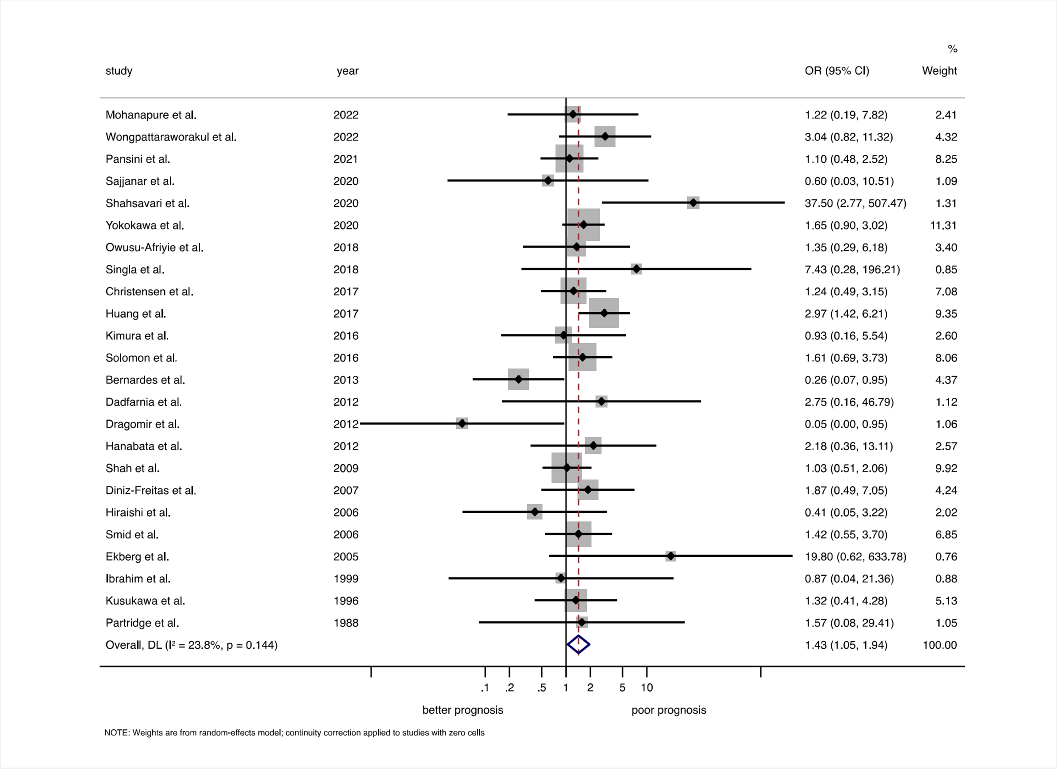

OSCC, oral squamous cell carcinoma; OR, odds ratio; CI, confidence intervals. Random-effects model, inverse-variance weighting (based on the DerSimonian and Laird method). A OR > 1 suggests that EGFR overexpression is associated with a higher grade. Diamonds indicate the pooled ORs with their corresponding 95% CIs.

9. Analysis of small-study effects

9.1 EGFR overexpression and overall survival in OSCC

Figure S20. A funnel plot of estimated effect size against their standard errors, graphically representing the analysis of small-study effects on the association between EGFR overexpression and overall survival in OSCC.

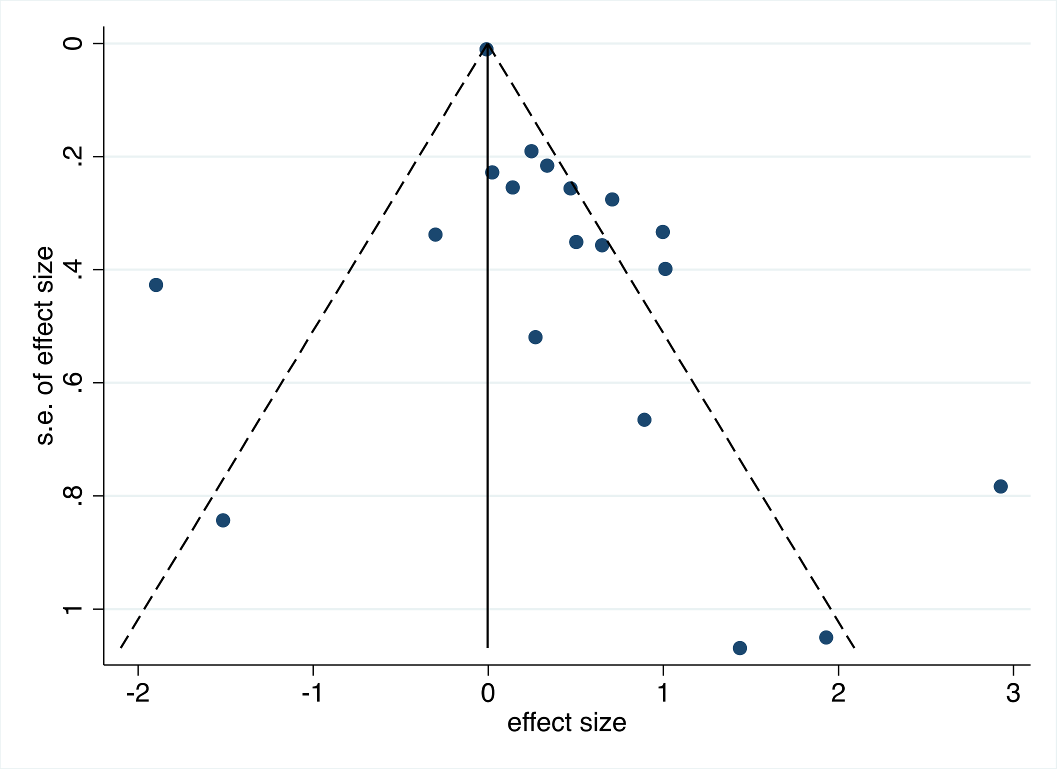

s.e., standard error. The black vertical line corresponds to the pooled estimated prevalence. The two diagonal intermittent lines represent the pseudo-95% confidence interval. The blue circles represent the estimates from primary-level studies.

9.2 EGFR overexpression and DFS in OSCC

Figure S21. A funnel plot of estimated effect size against their standard errors, graphically representing the analysis of small-study effects on the association between EGFR overexpression and DFS in OSCC.

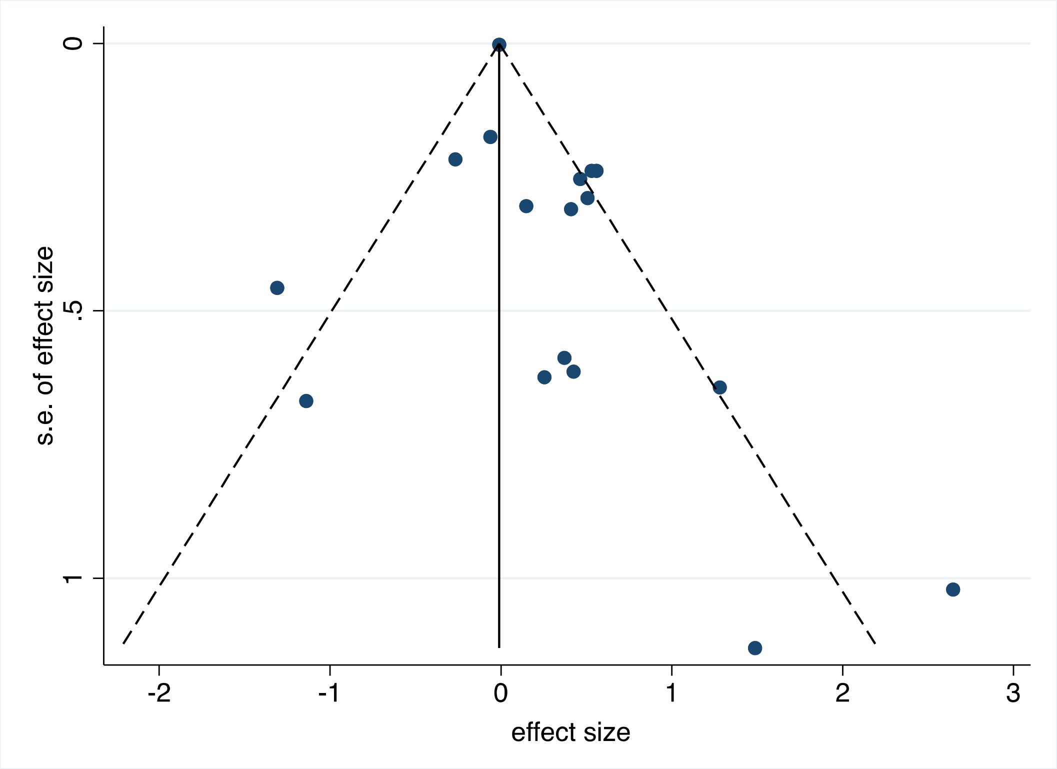

DFS, disease-free survival; s.e., standard error; HR, hazard ratio; log, natural logarithm (i.e., log base e). The black vertical line corresponds to the pooled estimated prevalence. The two diagonal intermittent lines represent the pseudo-95% confidence interval. The blue circles represent the estimates from primary-level studies.

9.3 EGFR overexpression and T status in OSCC

Figure S22. A funnel plot of estimated effect size against their standard errors, graphically representing the analysis of small-study effects on the association between EGFR overexpression and T status in OSCC.

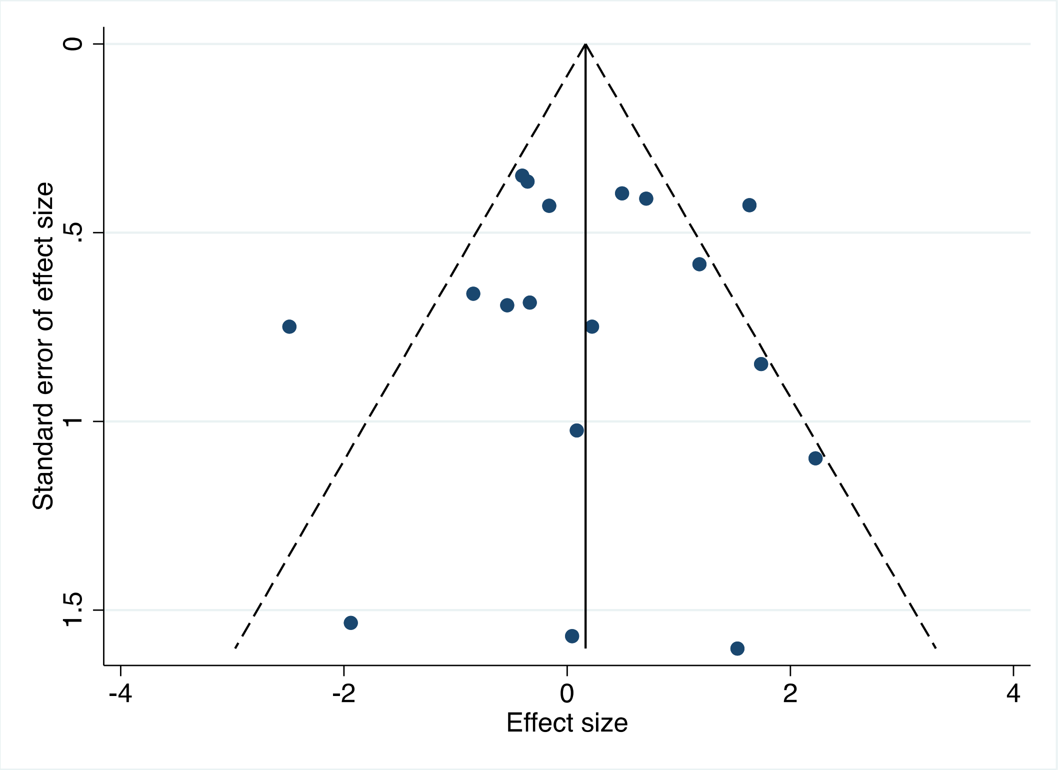

SE, standard error. The black vertical line corresponds to the pooled estimated prevalence. The two diagonal intermittent lines represent the pseudo-95% confidence interval. The blue circles represent the estimates from primary-level studies.

9.4 EGFR overexpression and N status in OSCC

Figure S23. A funnel plot of estimated effect size against their standard errors, graphically representing the analysis of small-study effects on the association between EGFR overexpression and N status in OSCC.

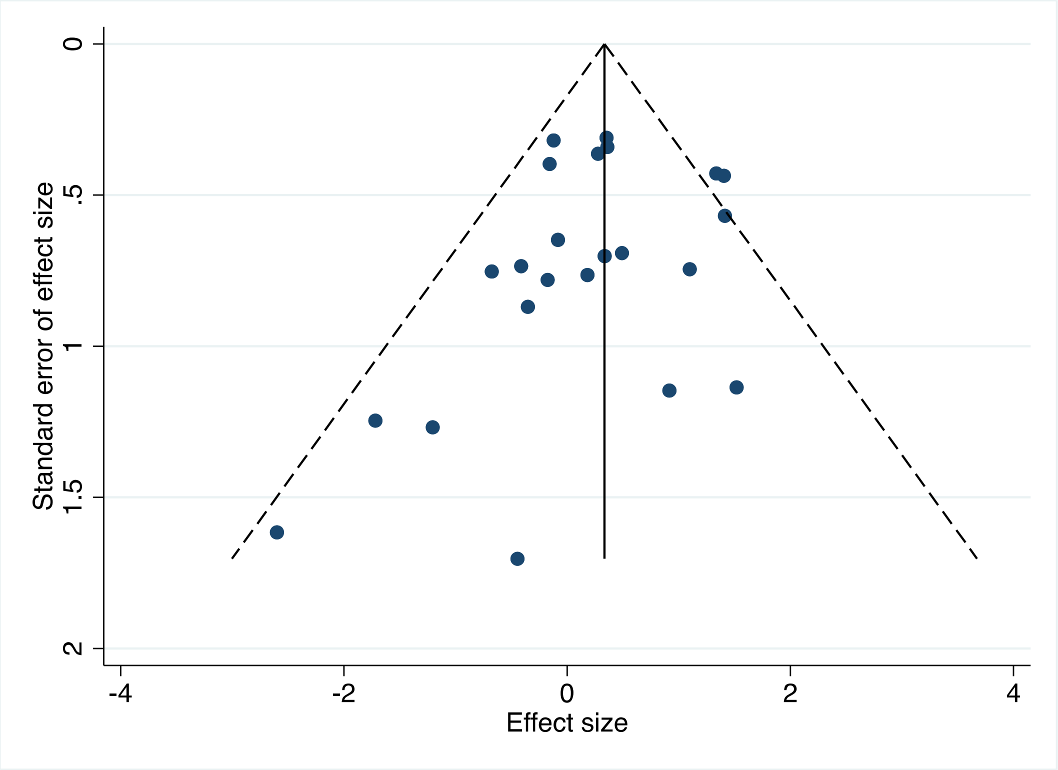

SE, standard error. The black vertical line corresponds to the pooled estimated prevalence. The two diagonal intermittent lines represent the pseudo-95% confidence interval. The blue circles represent the estimates from primary-level studies.

9.5 EGFR overexpression and clinical stage in OSCC

Figure S24. A funnel plot of estimated effect size against their standard errors, graphically representing the analysis of small-study effects on the association between EGFR overexpression and clinical stage in OSCC.

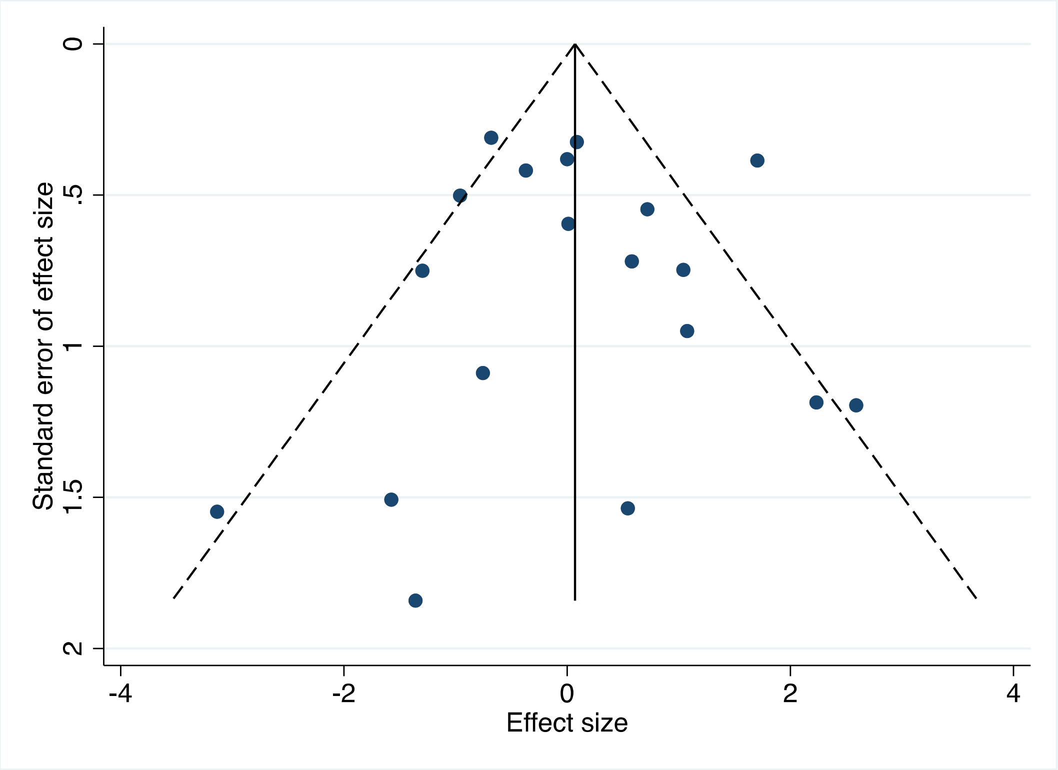

SE, standard error. The black vertical line corresponds to the pooled estimated prevalence. The two diagonal intermittent lines represent the pseudo-95% confidence interval. The blue circles represent the estimates from primary-level studies.

9.6 EGFR overexpression and histological grade in OSCC

Figure S25. A funnel plot of effect size against their standard errors, graphically representing the analysis of small-study effects on the association between EGFR overexpression and histological grade in OSCC.

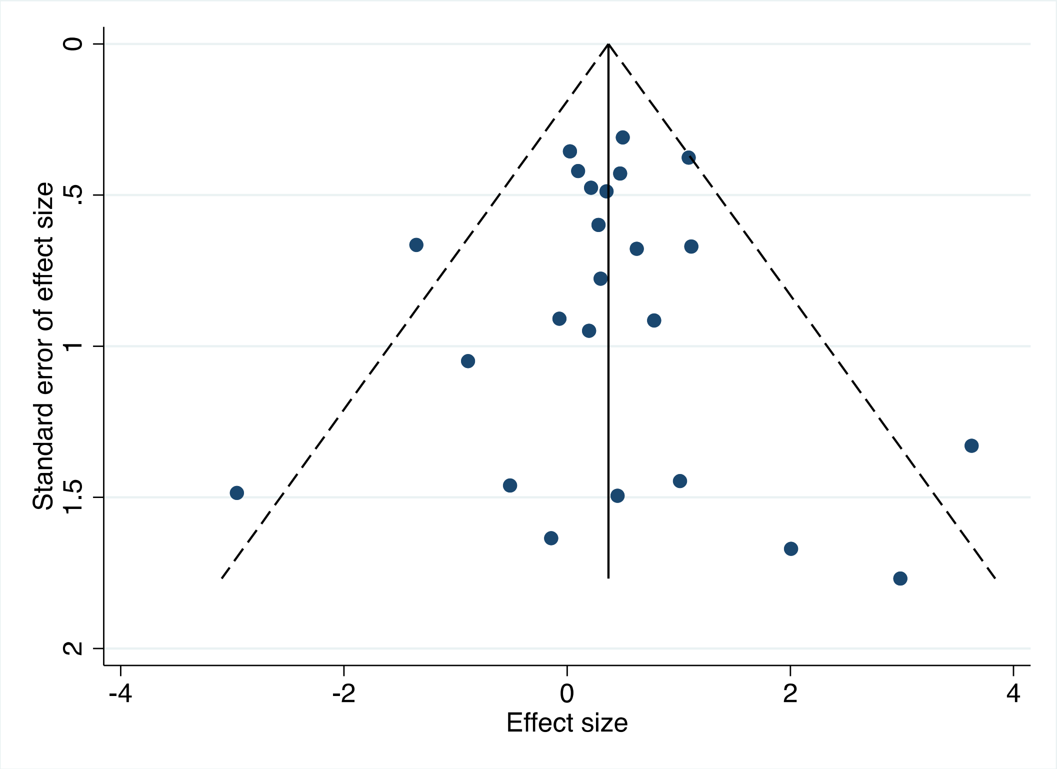

SE, standard error. The black vertical line corresponds to the pooled estimated prevalence. The two diagonal intermittent lines represent the pseudo-95% confidence interval. The blue circles represent the estimates from primary-level studies.

10. List of full-text articles excluded with reasons

Lack of essential data (n = 91) [1-91]:

1. Adduri R Sr, Kotapalli V, Gupta NA, Gowrishankar S, Srinivasulu M, Ali MM, Rao S, Uppin SG, Nayak UK, Dhagam S, Chigurupati MV, Bashyam MD. P53 nuclear stabilization is associated with FHIT loss and younger age of onset in squamous cell carcinoma of oral tongue. BMC Clin Pathol. 2014 Aug 9;14:37. doi: 10.1186/1472- 6890-14-37. PMID: 25152695; PMCID: PMC4141988.
2. Bandrés E, Barricarte R, Cantero C, Honorato B, Malumbres R, Zárate R, Alcalde J, García-Foncillas J. Epidermal growth factor receptor (EGFR) polymorphisms and survival in head and neck cancer patients. Oral Oncol. 2007 Aug;43(7):713-9. doi: 10.1016/j.oraloncology.2006.09.002. Epub 2006 Nov 16. PMID: 17112774.
3. Baschnagel AM, Tonlaar N, Eskandari M, Kumar T, Williams L, Hanna A, Pruetz BL, Wilson GD. Combined CD44, c-MET, and EGFR expression in p16-positive and p16-negative head and neck squamous cell carcinomas. J Oral Pathol Med. 2017 Mar;46(3):208-213. doi: 10.1111/jop.12478. Epub 2016 Jul 21. PMID: 27442811.
4. Beck C, Piontek G, Haug A, Bas M, Knopf A, Stark T, Mißlbeck M, Rudelius M, Reiter R, Brandstetter M, Pickhard A. The kallikrein-kinin-system in head and neck squamous cell carcinoma (HNSCC) and its role in tumour survival, invasion, migration and response to radiotherapy. Oral Oncol. 2012 Dec;48(12):1208-19. doi: 10.1016/j.oraloncology.2012.06.001. Epub 2012 Jun 27. PMID: 22748451.
5. Bergler W, Bier H, Ganzer U. The expression of epidermal growth factor receptors in the oral mucosa of patients with oral cancer. Arch Otorhinolaryngol. 1989;246(3):121-5. doi: 10.1007/BF00456651. PMID: 2474288.
6. Carlson AL, Gillenwater AM, Williams MD, El-Naggar AK, Richards-Kortum RR. Confocal microscopy and molecular-specific optical contrast agents for the detection of oral neoplasia. Technol Cancer Res Treat. 2007 Oct;6(5):361-74. doi: 10.1177/153303460700600501. PMID: 17877424; PMCID: PMC2772068.
7. Cavicchioli Buim ME, Gurgel CA, Gonçalves Ramos EA, Lourenço SV, Soares FA. Activation of sonic hedgehog signaling in oral squamous cell carcinomas: a preliminary study. Hum Pathol. 2011 Oct;42(10):1484-90. doi: 10.1016/j.humpath.2010.12.015. Epub 2011 Apr 14. PMID: 21496866.
8. Chu PY, Liou JH, Lin YM, Chen CJ, Chen MK, Lin SH, Yeh CM, Wang HK, Maa MC, Leu TH, Chang NW, Hsu NC, Yeh KT. Expression of Eps8 correlates with poor survival in oral squamous cell carcinoma. Asia Pac J Clin Oncol. 2012 Dec;8(4):e77- 81. doi: 10.1111/j.1743-7563.2011.01459.x. Epub 2012 Jan 12. PMID: 22897151.
9. Cohen EE, Lingen MW, Zhu B, Zhu H, Straza MW, Pierce C, Martin LE, Rosner MR. Protein kinase C zeta mediates epidermal growth factor-induced growth of head and neck tumor cells by regulating mitogen-activated protein kinase. Cancer Res. 2006 Jun 15;66(12):6296-303. doi: 10.1158/0008-5472.CAN-05-3139. PMID: 16778206.
10. Cohen ER, Reis IM, Gomez C, Pereira L, Freiser ME, Hoosien G, Franzmann EJ. Immunohistochemistry Analysis of CD44, EGFR, and p16 in Oral Cavity and Oropharyngeal Squamous Cell Carcinoma. Otolaryngol Head Neck Surg. 2017 Aug;157(2):239-251. doi: 10.1177/0194599817700371. Epub 2017 Apr 11. PMID: 28397583.
11. Cortelazzi B, Verderio P, Ciniselli CM, Pizzamiglio S, Bossi P, Gloghini A, Gualeni AV, Volpi CC, Locati L, Pierotti MA, Licitra L, Pilotti S, Perrone F. Receptor tyrosine kinase profiles and human papillomavirus status in oropharyngeal squamous cell carcinoma. J Oral Pathol Med. 2015 Oct;44(9):734-45. doi: 10.1111/jop.12301. Epub 2014 Dec 14. PMID: 25495427.
12. da Costa AA, D'Almeida Costa F, Ribeiro AR, Guimarães AP, Chinen LT, Lopes CA, de Lima VC. Low PTEN expression is associated with worse overall survival in head and neck squamous cell carcinoma patients treated with chemotherapy and cetuximab. Int J Clin Oncol. 2015 Apr;20(2):282-9. doi: 10.1007/s10147-014-0707-1. Epub 2014 May 27. PMID: 24858479.
13. da Silva Baumgart C, da Silva Lauxen I, Filho MS, de Quadros OF. Epidermal growth factor receptor distribution in pericoronal follicles: relationship with the origin of odontogenic cysts

and tumors. Oral Surg Oral Med Oral Pathol Oral Radiol Endod. 2007 Feb;103(2):240-5. doi: 10.1016/j.tripleo.2005.11.009. Epub 2006 Mar 24. PMID: 17234542.

14. Del Sordo R, Angiero F, Bellezza G, Cavaliere A, Mameli MG, Stefani M, Dessy E, Sidoni A. HER family receptors expression in squamous cell carcinoma of the tongue: study of the possible prognostic and biological significance. J Oral Pathol Med. 2010 Jan;39(1):79-86. doi: 10.1111/j.1600-0714.2009.00815.x. Epub 2009 Aug 18. PMID: 19691460.
15. Doescher J, Piontek G, Wirth M, Bettstetter M, Schlegel J, Haller B, Brockhoff G, Reiter R, Pickhard A. Epstein-Barr virus infection is strictly associated with the metastatic spread of sinonasal squamous-cell carcinomas. Oral Oncol. 2015 Oct;51(10):929-34. doi: 10.1016/j.oraloncology.2015.07.008. Epub 2015 Aug 10. PMID: 26272275.
16. Duray A, Descamps G, Decaestecker C, Remmelink M, Sirtaine N, Lechien J, Ernoux-Neufcoeur P, Bletard N, Somja J, Depuydt CE, Delvenne P, Saussez S. Human papillomavirus DNA strongly correlates with a poorer prognosis in oral cavity carcinoma. Laryngoscope. 2012 Jul;122(7):1558-65. doi: 10.1002/lary.23298. Epub 2012 Apr 24. PMID: 22532307.
17. Elango KJ, Suresh A, Erode EM, Subhadradevi L, Ravindran HK, Iyer SK, Iyer SK, Kuriakose MA. Role of human papilloma virus in oral tongue squamous cell carcinoma. Asian Pac J Cancer Prev. 2011;12(4):889-96. PMID: 21790221.
18. Fakurnejad S, van Keulen S, Nishio N, Engelen M, van den Berg NS, Lu G, Birkeland A, Baik F, Colevas AD, Rosenthal EL, Martin BA. Fluorescence molecular imaging for identification of high-grade dysplasia in patients with head and neck cancer. Oral Oncol. 2019 Oct;97:50-55. doi: 10.1016/j.oraloncology.2019.08.008. Epub 2019 Aug 12. PMID: 31421471; PMCID: PMC6907742.
19. Fong Y, Chou SJ, Hung KF, Wu HT, Kao SY. An investigation of the differential expression of Her2/neu gene expression in normal oral mucosa, epithelial dysplasia, and oral squamous cell carcinoma in Taiwan. J Chin Med Assoc. 2008 Mar;71(3):123-7. doi: 10.1016/S1726-4901(08)70003-0. PMID: 18364263.
20. Freier K, Bosch FX, Flechtenmacher C, Devens F, Benner A, Lichter P, Joos S, Hofele C. Distinct site-specific oncoprotein overexpression in head and neck squamous cell carcinoma: a tissue microarray analysis. Anticancer Res. 2003 Sep- Oct;23(5A):3971-7. PMID: 14666705.
21. Freier K, Joos S, Flechtenmacher C, Devens F, Benner A, Bosch FX, Lichter P, Hofele C. Tissue microarray analysis reveals site-specific prevalence of oncogene amplifications in head and neck squamous cell carcinoma. Cancer Res. 2003 Mar 15;63(6):1179-82. PMID: 12649172.
22. Frohwitter G, Buerger H, Korsching E, van Diest PJ, Kleinheinz J, Fillies T. Site-specific gene expression patterns in oral cancer. Head Face Med. 2017 May 10;13(1):6. doi: 10.1186/s13005-017-0138-0. PMID: 28486965; PMCID: PMC5424406.
23. Gao L, Zhang W, Zhong WQ, Liu ZJ, Li HM, Yu ZL, Zhao YF. Tumor associated macrophages induce epithelial to mesenchymal transition via the EGFR/ERK1/2 pathway in head and neck squamous cell carcinoma. Oncol Rep. 2018 Nov;40(5):2558-2572. doi: 10.3892/or.2018.6657. Epub 2018 Aug 17. PMID: 30132555; PMCID: PMC6151899.
24. Grimm M, Iftner T, Altaki H, Iftner A, Peters JP, Munz A, Reinert S. Detection of mutation-specific epidermal growth factor receptor (E746-A750del) and lack of detection of human papillomavirus in oral squamous cell carcinoma. Int J Oral Maxillofac Surg. 2014 Oct;43(10):1199-205. doi: 10.1016/j.ijom.2014.04.006. Epub 2014 May 10. PMID: 24818747.
25. Hinsley EE, Hunt S, Hunter KD, Whawell SA, Lambert DW. Endothelin-1 stimulates motility of head and neck squamous carcinoma cells by promoting stromal-epithelial interactions. Int J Cancer. 2012 Jan 1;130(1):40-7. doi: 10.1002/ijc.25968. Epub 2011 Apr 13. PMID: 21491424.
26. Hoesli R, Birkeland AC, Rosko AJ, Issa M, Chow KL, Michmerhuizen NL, Mann JE, Chinn SB, Shuman AG, Prince ME, Wolf GT, Bradford CR, McHugh JB, Brenner JC, Spector ME. Proportion of CD4 and CD8 tumor infiltrating lymphocytes predicts survival in persistent/recurrent laryngeal squamous cell carcinoma. Oral Oncol. 2018 Feb;77:83-89. doi: 10.1016/j.oraloncology.2017.12.003. Epub 2017 Dec 23. PMID: 29362129; PMCID: PMC5897103.

27. Hoffmann TK, Balló H, Braunstein S, Van Lierop A, Wagenmann M, Bier H. Serum level and tissue expression of c-erbB-1 and c-erbB-2 proto-oncogene products in patients with squamous cell carcinoma of the head and neck. Oral Oncol. 2001 Jan;37(1):50-6. doi: 10.1016/s1368-8375(00)00056-7. PMID: 11120483.
28. Huang HJ, Ping FY, Hu JA, Zhao SF. Downregulation of Notch1 and its potential correlation with epidermal growth factor receptor signalling in tongue squamous cell carcinoma. Br J Oral Maxillofac Surg. 2010 Jan;48(1):30-6. doi: 10.1016/j.bjoms.2009.03.008. Epub 2009 Apr 23. PMID: 19398252.
29. Huang WC, Chan SH, Jang TH, Chang JW, Ko YC, Yen TC, Chiang SL, Chiang WF, Shieh TY, Liao CT, Juang JL, Wang HC, Cheng AJ, Lu YC, Wang LH. miRNA-491- 5p and GIT1 serve as modulators and biomarkers for oral squamous cell carcinoma invasion and metastasis. Cancer Res. 2014 Feb 1;74(3):751-64. doi: 10.1158/0008- 5472.CAN-13-1297. Epub 2013 Dec 12. PMID: 24335959.
30. Hwang YS, Lee SK, Park KK, Chung WY. Secretion of IL-6 and IL-8 from lysophosphatidic acid-stimulated oral squamous cell carcinoma promotes osteoclastogenesis and bone resorption. Oral Oncol. 2012 Jan;48(1):40-8. doi: 10.1016/j.oraloncology.2011.08.022. Epub 2011 Sep 16. PMID: 21925926.
31. Jiang Y, Liao L, Shrestha C, Ji S, Chen Y, Peng J, Wang L, Liao E, Xie Z. Reduced expression of E-cadherin and p120-catenin and elevated expression of PLC- γ1 and PIKE are associated with aggressiveness of oral squamous cell carcinoma. Int J Clin Exp Pathol. 2015 Aug 1;8(8):9042-51. PMID: 26464646; PMCID: PMC4583878.
32. Kannan S, Balaram P, Chandran GJ, Pillai MR, Mathew B, Nair MK. Co- expression of ras p21 and epidermal growth factor receptor during various stages of tumour progression in oral mucosa. Tumour Biol. 1994;15(2):73-81. doi: 10.1159/000217877. PMID: 8184255.
33. Kannan S, Chandran GJ, Balaram P, Chidambaram S, Nair MK. Potential biological markers for the staging of tumor progression in oral mucosa: a multivariate analysis. Int J Biol Markers. 1996 Apr-Jun;11(2):67-76. doi: 10.1177/172460089601100202. PMID: 8776606.
34. Katz J, Jakymiw A, Ducksworth MK, Stewart CM, Bhattacharyya I, Cha S, Chan EK. CIP2A expression and localization in oral carcinoma and dysplasia. Cancer Biol Ther. 2010 Oct 1;10(7):694-9. doi: 10.4161/cbt.10.7.12895. PMID: 21068540; PMCID: PMC3230513.
35. Kawamoto T, Takahashi K, Nishi M, Kimura T, Matsumura T, Taniguchi S. Quantitative assay of epidermal growth factor receptor in human squamous cell carcinomas of the oral region by an avidin-biotin method. Jpn J Cancer Res. 1991 Apr;82(4):403-10. doi: 10.1111/j.1349-7006.1991.tb01863.x. PMID: 1904421; PMCID: PMC5918449.
36. Kearsley JH, Furlong KL, Cooke RA, Waters MJ. An immunohistochemical assessment of cellular proliferation markers in head and neck squamous cell cancers. Br J Cancer. 1990 Jun;61(6):821-7. doi: 10.1038/bjc.1990.184. PMID: 2372483; PMCID: PMC1971683.
37. Kim HS, Kwon HJ, Jung I, Yun MR, Ahn MJ, Kang BW, Sun JM, Kim SB, Yoon DH, Park KU, Lee SH, Koh YW, Kim SH, Choi EC, Koo DH, Sohn JH, Kim B, Kwon NJ, Yun HJ, Lee MG, Lee JH, Kim TM, Kim HR, Kim JH, Paik S, Cho BC. Phase II clinical and exploratory biomarker study of dacomitinib in patients with recurrent and/or metastatic squamous cell carcinoma of head and neck. Clin Cancer Res. 2015 Feb 1;21(3):544-52. doi: 10.1158/1078-0432.CCR-14-1756. Epub 2014 Nov 25. PMID: 25424851.
38. Kiyota A, Shintani S, Mihara M, Nakahara Y, Ueyama Y, Matsumura T, Todd R, Wong DT. Expression of a truncated epidermal growth factor receptor in oral squamous cell carcinomas. Cancer Lett. 2000 Dec 8;161(1):9-15. doi: 10.1016/s0304-3835(00)00573-5. PMID: 11078908.
39. Kongkaew T, Aung WPP, Supanchart C, Makeudom A, Langsa-Ard S, Sastraruji T, Chaiyarit P, Krisanaprakornkit S. O-GlcNAcylation in oral squamous cell carcinoma. J Oral Pathol Med. 2018 Mar;47(3):260-267. doi: 10.1111/jop.12680. Epub 2018 Jan 30. PMID: 29327476.
40. Kouhsoltani M, Aghbali A, Shokoohi B, Ahmadzadeh R. Molecular Targeting of Her-2/neu Protein Is Not Recommended as an Adjuvant Therapy in Oral Squamous Cell Carcinoma and

Oral Lichen Planus. Adv Pharm Bull. 2015 Dec;5(Suppl 1):649-52. doi: 10.15171/apb.2015.088. Epub 2015 Dec 31. PMID: 26793611; PMCID: PMC4708036.

41. Lauxen IS, Oliveira MG, Rados PV, Lingen MW, Nör JE, Sant'ana Filho M. Immunoprofiling of oral squamous cell carcinomas reveals high p63 and survivin expression. Oral Dis. 2014 Apr;20(3):e76-80. doi: 10.1111/odi.12136. Epub 2013 Jun 6. PMID: 23738964.
42. Lindell Jonsson E, Nylander K, Hallén L, Laurell G. Immunohistochemical analysis of EGFR and hyaluronan in tongue cancer and the development of regional recurrence in patients initially diagnosed N0. Acta Otolaryngol. 2017 Aug;137(8):877- 882. doi: 10.1080/00016489.2017.1292049. Epub 2017 Mar 30. PMID: 28355940.
43. Lu WC, Kao SY, Yang CC, Tu HF, Wu CH, Chang KW, Lin SC. EGF up-regulates miR-31 through the C/EBPβ signal cascade in oral carcinoma. PLoS One. 2014 Sep 17;9(9):e108049. doi: 10.1371/journal.pone.0108049. PMID: 25229239; PMCID: PMC4168139.
44. Mak MP, William WN Jr. Targeting the epidermal growth factor receptor for head and neck cancer chemoprevention. Oral Oncol. 2014 Oct;50(10):918-23. doi: 10.1016/j.oraloncology.2013.12.024. Epub 2014 Jan 10. PMID: 24412287; PMCID: PMC4090290.
45. Martín-Ezquerra G, Salgado R, Toll A, Gilaberte M, Baró T, Alameda Quitllet F, Yébenes M, Solé F, Garcia-Muret M, Espinet B, Pujol RM. Multiple genetic copy number alterations in oral squamous cell carcinoma: study of MYC, TP53, CCDN1, EGFR and ERBB2 status in primary and metastatic tumours. Br J Dermatol. 2010 Nov;163(5):1028-35. doi: 10.1111/j.1365-2133.2010.09947.x. PMID: 20662834.
46. McIntyre JB, Bose P, Klimowicz AC, Brockton NT, Petrillo S, Matthews W, Easaw J, Magliocco A, Dort JC. Specific and sensitive hydrolysis probe-based real- time PCR detection of epidermal growth factor receptor variant III in oral squamous cell carcinoma. PLoS One. 2012;7(2):e31723. doi: 10.1371/journal.pone.0031723. Epub 2012 Feb 16. PMID: 22359620; PMCID: PMC3280998.
47. Mehta A, Chowdhary M, Sinha R. Immunoscoring of epidermal growth factor receptor expression in recurrent cases of oral squamous cell carcinoma. J Oral Pathol Med. 2015 Nov;44(10):818-22. doi: 10.1111/jop.12303. Epub 2015 Jan 12. PMID: 25581523.
48. Mehta DT, Annamalai T, Ramanathan A. Lack of mutations in protein tyrosine kinase domain coding exons 19 and 21 of the EGFR gene in oral squamous cell carcinomas. Asian Pac J Cancer Prev. 2014;15(11):4623-7. doi: 10.7314/apjcp.2014.15.11.4623. PMID: 24969895.
49. Mendelsohn AH, Lai CK, Shintaku IP, Elashoff DA, Dubinett SM, Abemayor E, St John MA. Histopathologic findings of HPV and p16 positive HNSCC. Laryngoscope. 2010 Sep;120(9):1788-94. doi: 10.1002/lary.21044. PMID: 20803740.
50. Minabe M, Akiyama Y, Higa K, Tachikawa T, Takahashi S, Nomura T, Kouno M. A potential link between desmoglein 3 and epidermal growth factor receptor in oral squamous cell carcinoma and its effect on cetuximab treatment efficacy. Exp Dermatol. 2019 May;28(5):614-617. doi: 10.1111/exd.13920. PMID: 30907457.
51. Miranda Galvis M, Freitas Jardim J, Kaminagakura E, Santos-Silva AR, Paiva Fonseca F, Paes Almeida O, Ajudarte Lopes M, Lópes Pinto C, Kowalski LP. Expression of cell cycle proteins according to HPV status in oral squamous cell carcinoma affecting young patients: a pilot study. Oral Surg Oral Med Oral Pathol Oral Radiol. 2018 Apr;125(4):317-325. doi: 10.1016/j.oooo.2018.01.003. Epub 2018 Feb 2. PMID: 29501355.
52. Miranda Galvis M, Santos-Silva AR, Freitas Jardim J, Paiva Fonseca F, Lopes MA, de Almeida OP, Lópes Pinto CA, Kaminagakura E, Sawazaki-Calone I, Speight PM, Kowalski LP. Different patterns of expression of cell cycle control and local invasion-related proteins in oral squamous cell carcinoma affecting young patients. J Oral Pathol Med. 2018 Jan;47(1):32-39. doi: 10.1111/jop.12601. Epub 2017 Aug 4. PMID: 28626969.
53. Mishra A, Bharti AC, Saluja D, Das BC. Transactivation and expression patterns of Jun and Fos/AP-1 super-family proteins in human oral cancer. Int J Cancer. 2010 Feb 15;126(4):819-29. doi: 10.1002/ijc.24807. PMID: 19653276.

54. Pardis S, Sardari Y, Ashraf MJ, Andisheh Tadbir A, Ebrahimi H, Purshahidi S, Khademi B, Fattahi MJ, Hamzavi M. Evaluation of Tissue Expression and Salivary Levels of HER2/neu in Patients with Head and Neck Squamous cell Carcinoma. Iran J Otorhinolaryngol. 2012 Fall;24(69):161-70. PMID: 24303404; PMCID: PMC3846196.
55. Partridge M, Green MR, Langdon JD, Feldmann M. Production of TGF-alpha and TGF-beta by cultured keratinocytes, skin and oral squamous cell carcinomas-- potential autocrine regulation of normal and malignant epithelial cell proliferation. Br J Cancer. 1989 Oct;60(4):542-8. doi: 10.1038/bjc.1989.310. PMID: 2478181; PMCID: PMC2247118.
56. Pectasides E, Rampias T, Sasaki C, Perisanidis C, Kouloulias V, Burtness B, Zaramboukas T, Rimm D, Fountzilas G, Psyrri A. Markers of epithelial to mesenchymal transition in association with survival in head and neck squamous cell carcinoma (HNSCC). PLoS One. 2014 Apr 10;9(4):e94273. doi: 10.1371/journal.pone.0094273. PMID: 24722213; PMCID: PMC3983114.
57. Perez A, Neskey DM, Wen J, Pereira L, Reategui EP, Goodwin WJ, Carraway KL, Franzmann EJ. CD44 interacts with EGFR and promotes head and neck squamous cell carcinoma initiation and progression. Oral Oncol. 2013 Apr;49(4):306- 13. doi: 10.1016/j.oraloncology.2012.11.009. Epub 2012 Dec 20. PMID: 23265944; PMCID: PMC3594090.
58. Perisanidis C, Savarese-Brenner B, Würger T, Wrba F, Huynh A, Schopper C, Kornek G, Selzer E, Ewers R, Psyrri A, Krainer M, Filipits M. HCRP1 expression status is a significant prognostic marker in oral and oropharyngeal cancer. Oral Dis. 2013 Mar;19(2):206-11. doi: 10.1111/j.1601-0825.2012.01972.x. Epub 2012 Aug 14. PMID: 22891969.
59. Precht C, Blessmann M, Kluwe L, Scheld T, Schön G, Henningsen A, Pflug C, Smeets R, Heiland M, Gröbe A. Lack of evidence for prognostic value of epidermal growth factor receptor intron-1 CA repeats for oral carcinomas. Eur J Oral Sci. 2017 Apr;125(2):95-101. doi: 10.1111/eos.12333. Epub 2017 Feb 2. PMID: 28150908.
60. Raulf N, Lucarelli P, Thavaraj S, Brown S, Vicencio JM, Sauter T, Tavassoli M. Annexin A1 regulates EGFR activity and alters EGFR-containing tumour-derived exosomes in head and neck cancers. Eur J Cancer. 2018 Oct;102:52-68. doi: 10.1016/j.ejca.2018.07.123. Epub 2018 Aug 21. PMID: 30142511.
61. Rosbach KJ, Williams MD, Gillenwater AM, Richards-Kortum RR. Optical molecular imaging of multiple biomarkers of epithelial neoplasia: epidermal growth factor receptor expression and metabolic activity in oral mucosa. Transl Oncol. 2012 Jun;5(3):160-71. doi: 10.1593/tlo.11310. Epub 2012 Jun 1. PMID: 22741035; PMCID: PMC3384270.
62. Ryu MH, Park JH, Park JE, Chung J, Lee CH, Park HR. Cobalt chloride stimulates phosphoinositide 3-kinase/Akt signaling through the epidermal growth factor receptor in oral squamous cell carcinoma. Biocell. 2010 Apr;34(1):15-21. PMID: 20506627.
63. Sales KU, Giudice FS, Castilho RM, Salles FT, Squarize CH, Abrahao AC, Pinto DS Jr. Cyclin D1-induced proliferation is independent of beta-catenin in head and neck cancer. Oral Dis. 2014 Apr;20(3):e42-8. doi: 10.1111/odi.12124. Epub 2013 Jun 4. PMID: 23730900.
64. Sardari Y, Pardis S, Tadbir AA, Ashraf MJ, Fattahi MJ, Ebrahimi H, Purshahidi S, Khademi B, Hamzavi M. HER2/neu expression in head and neck squamous cell carcinoma patients is not significantly elevated. Asian Pac J Cancer Prev. 2012;13(6):2891-6. doi: 10.7314/apjcp.2012.13.6.2891. PMID: 22938479.
65. Sarkis SA, Abdullah BH, Abdul Majeed BA, Talabani NG. Immunohistochemical expression of epidermal growth factor receptor (EGFR) in oral squamous cell carcinoma in relation to proliferation, apoptosis, angiogenesis and lymphangiogenesis. Head Neck Oncol. 2010 Jun 25;2:13. doi: 10.1186/1758-3284-2- 13. PMID: 20579333; PMCID: PMC2907378.
66. Shigeishi H, Higashikawa K, Hiraoka M, Fujimoto S, Mitani Y, Ohta K, Takechi M, Kamata N. Expression of epiregulin, a novel epidermal growth factor ligand associated with prognosis in human oral squamous cell carcinomas. Oncol Rep. 2008 Jun;19(6):1557-64. PMID: 18497965.
67. Shih CH, Chang YJ, Huang WC, Jang TH, Kung HJ, Wang WC, Yang MH, Lin MC, Huang SF, Chou SW, Chang E, Chiu H, Shieh TY, Chen YJ, Wang LH, Chen L. EZH2-mediated upregulation of ROS1 oncogene promotes oral cancer metastasis. Oncogene. 2017 Nov

23;36(47):6542-6554. doi: 10.1038/onc.2017.262. Epub 2017 Jul 31. PMID: 28759046; PMCID: PMC5702718.

68. Shirasuna K, Hayashido Y, Sugiyama M, Yoshioka H, Matsuya T. Immunohistochemical localization of epidermal growth factor (EGF) and EGF receptor in human oral mucosa and its malignancy. Virchows Arch A Pathol Anat Histopathol. 1991;418(4):349-53. doi: 10.1007/BF01600165. PMID: 2024456.
69. Son HK, Kim D, Lim Y, Kim J, Park I. A novel TGF-β receptor II mutation (I227T/N236D) promotes aggressive phenotype of oral squamous cell carcinoma via enhanced EGFR signaling. BMC Cancer. 2020 Nov 27;20(1):1163. doi: 10.1186/s12885-020-07669-5. PMID: 33246423; PMCID: PMC7694911.
70. Squarize CH, Castilho RM, Sriuranpong V, Pinto DS Jr, Gutkind JS. Molecular cross-talk between the NFkappaB and STAT3 signaling pathways in head and neck squamous cell carcinoma. Neoplasia. 2006 Sep;8(9):733-46. doi: 10.1593/neo.06274. PMID: 16984731; PMCID: PMC1584297.
71. Suwinski R, Jaworska M, Nikiel B, Grzegorz W, Bankowska-Wozniak M, Wojciech M, Krzysztof S, Dariusz L. Predicting the effect of accelerated fractionation in postoperative radiotherapy for head and neck cancer based on molecular marker profiles: data from a randomized clinical trial. Int J Radiat Oncol Biol Phys. 2010 Jun 1;77(2):438-46. doi: 10.1016/j.ijrobp.2009.05.021. Epub 2009 Sep 3. PMID: 19733016.
72. Szabó B, Nelhubel GA, Kárpáti A, Kenessey I, Jóri B, Székely C, Peták I, Lotz G, Hegedus Z, Hegedus B, Füle T, Döme B, Tímár J, Tóvári J. Clinical significance of genetic alterations and expression of epidermal growth factor receptor (EGFR) in head and neck squamous cell carcinomas. Oral Oncol. 2011 Jun;47(6):487-96. doi: 10.1016/j.oraloncology.2011.03.020. Epub 2011 Apr 16. PMID: 21498106.
73. Teng X, Ma L, Kyrkanides S, Raja V, Trochesset D, Brouxhon SM. Modulation of RTK by sEcad: a putative mechanism for oncogenicity in oropharyngeal SCCs. Oral Dis. 2015 Mar;21(2):185-94. doi: 10.1111/odi.12235. Epub 2014 Apr 1. PMID: 24612046.
74. Ulanovski D, Stern Y, Roizman P, Shpitzer T, Popovtzer A, Feinmesser R. Expression of EGFR and Cerb-B2 as prognostic factors in cancer of the tongue. Oral Oncol. 2004 May;40(5):532-7. doi: 10.1016/j.oraloncology.2003.11.004. PMID: 15006627.
75. Vainshtein JM, Spector ME, McHugh JB, Wong KK, Walline HM, Byrd SA, Komarck CM, Ibrahim M, Stenmark MH, Prince ME, Bradford CR, Wolf GT, McLean S, Worden FP, Chepeha DB, Carey T, Eisbruch A. Refining risk stratification for locoregional failure after chemoradiotherapy in human papillomavirus-associated oropharyngeal cancer. Oral Oncol. 2014 May;50(5):513-9. doi: 10.1016/j.oraloncology.2014.02.001. Epub 2014 Feb 22. PMID: 24565983; PMCID: PMC4086868.
76. Valiathan GM, Thenumgal SJ, Jayaraman B, Palaniyandi A, Ramkumar H, Jayakumar K, Bhaskaran S, Ramanathan A. Common docking domain mutation E322K of the ERK2 gene is infrequent in oral squamous cell carcinomas. Asian Pac J Cancer Prev. 2012;13(12):6155-7. doi: 10.7314/apjcp.2012.13.12.6155. PMID: 23464422.
77. van Keulen S, Nishio N, Fakurnejad S, van den Berg NS, Lu G, Birkeland A, Martin BA, Forouzanfar T, Colevas AD, Rosenthal EL. Intraoperative Tumor Assessment Using Real-Time Molecular Imaging in Head and Neck Cancer Patients. J Am Coll Surg. 2019 Dec;229(6):560-567.e1. doi: 10.1016/j.jamcollsurg.2019.09.007. Epub 2019 Sep 27. PMID: 31568855.
78. van Schaik JE, Hanemaaijer SH, Halmos GB, Witjes MJH, van der Laan BFAM, van der Vegt B, Plaat BEC. Glycoprotein Nonmetastatic Melanoma Protein B as Potential Imaging Marker in Posttherapeutic Metastatic Head and Neck Cancer. Otolaryngol Head Neck Surg. 2020 Dec;163(6):1202-1208. doi: 10.1177/0194599820932869. Epub 2020 Jun 30. PMID: 32600105; PMCID: PMC7708663.
79. Varun C, Dineshkumar T, Jayant VS, Rameshkumar A, Rajkumar K, Rajashree P, Mathew J, Arunvignesh RK. Salivary Her2/neu Levels in Differentiation of Oral Premalignant Disorders

and Oral Squamous Cell Carcinomas. Asian Pac J Cancer Prev. 2015;16(14):5773-7. doi: 10.7314/apjcp.2015.16.14.5773. PMID: 26320450.
80. Wang YP, Liu IJ, Chen KC, Wu HC. NOTCH1 signaling promotes protein stability of HER3 through the AKT pathway in squamous cell carcinoma of head and neck. Oncogenesis. 2021 Aug 31;10(8):59. doi: 10.1038/s41389-021-00348-5. PMID: 34465724; PMCID: PMC8408252.
81. Young MR, Neville BW, Chi AC, Lathers DM, Boyd Gillespie M, Day TA. Oral premalignant lesions induce immune reactivity to both premalignant oral lesions and head and neck squamous cell carcinoma. Cancer Immunol Immunother. 2007 Jul;56(7):1077-86. doi: 10.1007/s00262-006-0242-7. Epub 2006 Nov 10. PMID: 17096152.
82. Zhang L, Liu SL, Ye WM, Zheng J. STAT3 is a key pathway in primary intraosseous squamous cell carcinoma arising from an odontogenic keratocyst. Am J Transl Res. 2015 Oct 15;7(10):1860-9. PMID: 26692930; PMCID: PMC4656763.
83. Hitt R, Ciruelos E, Amador ML, Benito A, Sanchez JJ, Ballestin C, Cortes- Funes H. Prognostic value of the epidermal growth factor receptor (EGRF) and p53 in advanced head and neck squamous cell carcinoma patients treated with induction chemotherapy. Eur J Cancer. 2005 Feb;41(3):453-60. doi: 10.1016/j.ejca.2004.10.014. PMID: 15691646.
84. Kejner AE, Burch MB, Sweeny L, Rosenthal EL. Bone morphogenetic protein 6 expression in oral cavity squamous cell cancer is associated with bone invasion. Laryngoscope. 2013 Dec;123(12):3061-5. doi: 10.1002/lary.24267. Epub 2013 Jul 1. PMID: 23775772; PMCID: PMC3805669.
85. Odajima T, Sasaki Y, Tanaka N, Kato-Mori Y, Asanuma H, Ikeda T, Satoh M, Hiratsuka H, Tokino T, Sawada N. Abnormal beta-catenin expression in oral cancer with no gene mutation: correlation with expression of cyclin D1 and epidermal growth factor receptor, Ki-67 labeling index, and clinicopathological features. Hum Pathol. 2005 Mar;36(3):234-41. doi: 10.1016/j.humpath.2004.12.009. PMID: 15791567.
86. Ongkeko WM, Altuna X, Weisman RA, Wang-Rodriguez J. Expression of protein tyrosine kinases in head and neck squamous cell carcinomas. Am J Clin Pathol. 2005 Jul;124(1):71-6. doi: 10.1309/BTLN5WTMJ3PCNRRC. PMID: 15923166.
87. Pickering CR, Shah K, Ahmed S, Rao A, Frederick MJ, Zhang J, Unruh AK, Wang J, Ginsberg LE, Kumar AJ, Myers JN, Hamilton JD. CT imaging correlates of genomic expression for oral cavity squamous cell carcinoma. AJNR Am J Neuroradiol. 2013 Sep;34(9):1818-22. doi: 10.3174/ajnr.A3635. Epub 2013 Jun 13. PMID: 23764725; PMCID: PMC7965627.
88. Putti TC, To KF, Hsu HC, Chan AT, Lai GM, Tse G, Lee YS, Whang-Peng J, Millward M, Lin L, Lin X, Lee CS. Expression of epidermal growth factor receptor in head and neck cancers correlates with clinical progression: a multicentre immunohistochemical study in the Asia-Pacific region. Histopathology. 2002 Aug;41(2):144-51. doi: 10.1046/j.1365-2559.2002.01436.x. PMID: 12147092.
89. Rautava J, Jee KJ, Miettinen PJ, Nagy B, Myllykangas S, Odell EW, Soukka T, Morgan PR, Heikinheimo K. ERBB receptors in developing, dysplastic and malignant oral epithelia. Oral Oncol. 2008 Mar;44(3):227-35. doi: 10.1016/j.oraloncology.2007.02.012. Epub 2007 Jun 28. PMID: 17604679.
90. Schartinger VH, Kacani L, Andrle J, Schwentner I, Wurm M, Obrist P, Oberaigner W, Sprinzl GM. Pharmacodiagnostic value of the HER family in head and neck squamous cell carcinoma. ORL J Otorhinolaryngol Relat Spec. 2004;66(1):21-6. doi: 10.1159/000077229. PMID: 15103197.
91. Silva SD, Cunha IW, Younes RN, Soares FA, Kowalski LP, Graner E. ErbB receptors and fatty acid synthase expression in aggressive head and neck squamous cell carcinomas. Oral Dis. 2010 Nov;16(8):774-80. doi: 10.1111/j.1601- 0825.2010.01687.x. PMID: 20604875.

No clinicopathological outcomes (n = 31) [1-31]**:**

1. Alsahafi EN, Thavaraj S, Sarvestani N, Novoplansky O, Elkabets M, Ayaz B, Tavassoli M, Legends MF. EGFR overexpression increases radiotherapy response in HPV-positive head and neck cancer through inhibition of DNA damage repair and HPV E6 downregulation. Cancer Lett. 2021 Feb 1;498:80-97. doi: 10.1016/j.canlet.2020.10.035. Epub 2020 Oct 31. PMID: 33137407.
2. Bei R, Pompa G, Vitolo D, Moriconi E, Ciocci L, Quaranta M, Frati L, Kraus MH, Muraro R. Co-localization of multiple ErbB receptors in stratified epithelium of oral squamous cell carcinoma. J Pathol. 2001 Oct;195(3):343-8. doi: 10.1002/path.965. PMID: 11673832.
3. Buranaphatthana W, Wu S, Makeudom A, Sastraruji T, Supanchart C, Krisanaprakornkit S. Involvement of the A disintegrin and metalloproteinase 9 in oral cancer cell invasion. Eur J Oral Sci. 2021 Jun;129(3):e12775. doi: 10.1111/eos.12775. Epub 2021 Mar 30. PMID: 33786875.
4. Chang KY, Tsai SY, Chen SH, Tsou HH, Yen CJ, Liu KJ, Fang HL, Wu HC, Chuang BF, Chou SW, Tang CK, Liu SY, Lu PJ, Yen CY, Chang JY. Dissecting the EGFR- PI3K-AKT pathway in oral cancer highlights the role of the EGFR variant III and its clinical relevance. J Biomed Sci. 2013 Jun 27;20(1):43. doi: 10.1186/1423-0127-20-43. PMID: 23806066; PMCID: PMC3710269.
5. Choi YS, Park M, Kwon HJ, Koh YW, Lee SK, Kim J. Human Papillomavirus and Epidermal Growth Factor Receptor in Oral Cavity and Oropharyngeal Squamous Cell Carcinoma: Correlation With Dynamic Contrast-Enhanced MRI Parameters. AJR Am J Roentgenol. 2016 Feb;206(2):408-13. doi: 10.2214/AJR.15.14713. PMID: 26797372.
6. Christensen ME, Therkildsen MH, Hansen BL, Albeck H, Hansen GN, Bretlau P. Epidermal growth factor receptor expression on oral mucosa dysplastic epithelia and squamous cell carcinomas. Eur Arch Otorhinolaryngol. 1992;249(5):243-7. doi: 10.1007/BF00714485. PMID: 1524803.
7. Concha-Benavente F, Srivastava RM, Trivedi S, Lei Y, Chandran U, Seethala RR, Freeman GJ, Ferris RL. Identification of the Cell-Intrinsic and -Extrinsic Pathways Downstream of EGFR and IFNγ That Induce PD-L1 Expression in Head and Neck Cancer. Cancer Res. 2016 Mar 1;76(5):1031-43. doi: 10.1158/0008-5472.CAN-15-2001. Epub 2015 Dec 16. PMID: 26676749; PMCID: PMC4775348.
8. Dasgupta S, Biswas N, Deb AR, Chakrabarti S. Is there any correlation between tumor infiltrating lymphocytes and expression of EGFR in squamous cell carcinoma of head and neck? A retrospective observational study. Indian J Pathol Microbiol. 2022 Apr-Jun;65(2):262-267. doi: 10.4103/IJPM.IJPM_1251_20. PMID: 35435357.
9. Favia G, Corsalini M, Iacobellis M, Maiorano E. Carcinoma squamoso su lichen ruber planus orale. Studio clinico-patologico ed immunoistochimico di 11 casi [Squamous cell carcinoma in oral lichen ruber planus. A clinico-pathological and immunohistochemical study of 11 cases]. Minerva Stomatol. 1994 Oct;43(10):479-91. Italian. PMID: 7700225.
10. Frohwitter G, Buerger H, VAN Diest PJ, Korsching E, Kleinheinz J, Fillies T. Cytokeratin and protein expression patterns in squamous cell carcinoma of the oral cavity provide evidence for two distinct pathogenetic pathways. Oncol Lett. 2016 Jul;12(1):107-113. doi: 10.3892/ol.2016.4588. Epub 2016 May 16. PMID: 27347109; PMCID: PMC4906805.
11. Kondo Y, Suzuki S, Ono S, Goto M, Miyabe S, Ogawa T, Tsuchida H, Ito H, Takahara T, Satou A, Tsuzuki T, Yoshikawa K, Ueda R, Nagao T. In Situ PD-L1 Expression in Oral Squamous Cell Carcinoma Is Induced by Heterogeneous Mechanisms among Patients. Int J Mol Sci. 2022 Apr 7;23(8):4077. doi: 10.3390/ijms23084077. PMID: 35456895; PMCID: PMC9029520.
12. Mestre JR, Subbaramaiah K, Sacks PG, Schantz SP, Tanabe T, Inoue H, Dannenberg AJ. Retinoids suppress epidermal growth factor-induced transcription of cyclooxygenase-2 in human oral squamous carcinoma cells. Cancer Res. 1997 Jul 15;57(14):2890-5. PMID: 9230197.
13. Méndez-Matías G, Velázquez-Velázquez C, Castro-Oropeza R, Mantilla- Morales A, Ocampo-Sandoval D, Burgos-González A, Heredia-Gutiérrez C, Alvarado- Cabrero I, Sánchez-Sandoval R, Barco-Bazán A, Chilaca-Rosas F, Piña-Sánchez P. Prevalence of HPV in Mexican Patients with Head and Neck Squamous Carcinoma and Identification of Potential Prognostic

Biomarkers. Cancers (Basel). 2021 Nov 9;13(22):5602. doi: 10.3390/cancers13225602. PMID: 34830760; PMCID: PMC8616077.
14. Nouri A, Cannell H, Onguti M, Tezabwala B, Oliver R. The possible relevance of the expression of MHC antigens and of EGF receptor in aggressive oral tumours. Int J Oncol. 1997 Jun;10(6):1217-22. doi: 10.3892/ijo.10.6.1217. PMID: 21533507.
15. Ohashi Y, Kumagai K, Miyata Y, Matsubara R, Kitaura K, Suzuki S, Hamada Y, Suzuki R. Overexpression of ErbB4 is an independent marker for lymph node metastasis in Japanese patients with oral squamous cell carcinoma. Oral Surg Oral Med Oral Pathol Oral Radiol. 2016 Sep;122(3):313-21. doi: 10.1016/j.oooo.2016.04.017. Epub 2016 May 11. Erratum in: Oral Surg Oral Med Oral Pathol Oral Radiol. 2016 Nov;122(5):658. PMID: 27444519.
16. Ohi K, Suzuki M, Koike S, Satake J, Matsu-Ura K, Takasaka T. [Expression of epidermal growth factor receptor in squamous cell carcinoma of the head and neck]. Nihon Jibiinkoka Gakkai Kaiho. 1993 Dec;96(12):2039-43. Japanese. doi: 10.3950/jibiinkoka.96.2039. PMID: 8295066.
17. Ren ZH, Lin CZ, Cao W, Yang R, Lu W, Liu ZQ, Chen YM, Yang X, Tian Z, Wang LZ, Li J, Wang X, Chen WT, Ji T, Zhang CP. CD73 is associated with poor prognosis in HNSCC. Oncotarget. 2016 Sep 20;7(38):61690-61702. doi: 10.18632/oncotarget.11435. PMID: 27557512; PMCID: PMC5308683.
18. Romanska HM, Potemski P, Collins SI, Williams H, Parmar S, Berditchevski F. Loss of CD151/Tspan24 from the complex with integrin α3β1 in invasive front of the tumour is a negative predictor of disease-free survival in oral squamous cell carcinoma. Oral Oncol. 2013 Mar;49(3):224-9. doi: 10.1016/j.oraloncology.2012.09.013. Epub 2012 Oct 23. PMID: 23099281.
19. Rosa AC, Soares AB, Santos FP, Furuse C, de Araújo VC. Immunoexpression of growth factors and receptors in polymorphous low-grade adenocarcinoma. J Oral Pathol Med. 2016 Aug;45(7):494-9. doi: 10.1111/jop.12402. Epub 2015 Dec 16. PMID: 26671844.
20. Rubin Grandis J, Melhem MF, Barnes EL, Tweardy DJ. Quantitative immunohistochemical analysis of transforming growth factor-alpha and epidermal growth factor receptor in patients with squamous cell carcinoma of the head and neck. Cancer. 1996 Sep 15;78(6):1284-92. doi: 10.1002/(SICI)1097- 0142(19960915)78:6<1284::AID-CNCR17>3.0.CO;2-X. PMID: 8826952.
21. Shiraishi N, Onda T, Hayashi K, Onidani K, Watanabe K, Sekikawa S, Shibahara T. Heat shock protein 90 as a molecular target for therapy in oral squamous cell carcinoma: Inhibitory effects of 17-DMAG and ganetespib on tumor cells. Oncol Rep. 2021 Feb;45(2):448-458. doi: 10.3892/or.2020.7873. Epub 2020 Nov 30. PMID: 33416122.
22. Szentkúti G, Dános K, Brauswetter D, Kiszner G, Krenács T, Csákó L, Répássy G, Tamás L. Correlations between prognosis and regional biomarker profiles in head and neck squamous cell carcinomas. Pathol Oncol Res. 2015 Jul;21(3):643-50. doi: 10.1007/s12253-014-9869-4. Epub 2014 Dec 31. PMID: 25547827.
23. Takes RP, Baatenburg de Jong RJ, Wijffels K, Schuuring E, Litvinov SV, Hermans J, van Krieken JH. Expression of genetic markers in lymph node metastases compared with their primary tumours in head and neck cancer. J Pathol. 2001 Jul;194(3):298-302. doi: 10.1002/1096-9896(200107)194:3<298::AID- PATH900>3.0.CO;2-Q. PMID: 11439361.
24. Tamás L, Szentkúti G, Eros M, Dános K, Brauswetter D, Szende B, Zsákovics I, Krenács T. Differential biomarker expression in head and neck cancer correlates with anatomical localization. Pathol Oncol Res. 2011 Sep;17(3):721-7. doi: 10.1007/s12253- 011-9376-9. Epub 2011 Apr 13. PMID: 21487776.
25. Tarle M, Müller D, Raguž M, Lukšić I. Significance of nuclear EGFR and ABCG2 expression in malignant transformation of oral potentially malignant disorders. Head Neck. 2022 Dec;44(12):2668-2677. doi: 10.1002/hed.27174. Epub 2022 Aug 19. PMID: 36325600.
26. van Oijen MG, Rijksen G, ten Broek FW, Slootweg PJ. Increased expression of epidermal growth factor receptor in normal epithelium adjacent to head and neck carcinomas independent

of tobacco and alcohol abuse. Oral Dis. 1998 Mar;4(1):4-8. doi: 10.1111/j.1601-0825.1998.tb00247.x. PMID: 9655037.
27. Wang WM, Yang SS, Shao SH, Nie HQ, Zhang J, Su T. Metformin Downregulates the Expression of Epidermal Growth Factor Receptor Independent of Lowering Blood Glucose in Oral Squamous Cell Carcinoma. Front Endocrinol (Lausanne). 2022 Feb 9;13:828608. doi: 10.3389/fendo.2022.828608. PMID: 35222283; PMCID: PMC8864766.
28. Zhao M, Fu XL, Lv H. [The expression of EGFR in oral lichen planus, squamous cell papilloma and squamous cell carcinoma]. Shanghai Kou Qiang Yi Xue. 2012 Dec;21(6):673-6. Chinese. PMID: 23364554.
29. Zhou Q, van den Berg NS, Kang W, Pei J, Nishio N, van Keulen S, Engelen MA, Lee YJ, Hom M, Vega Leonel JCM, Hart Z, Vogel H, Cayrol R, Martin BA, Roesner M, Shields G, Lui N, Gephart MH, Raymundo RC, Yi G, Granucci M, Grant GA, Li G, Rosenthal EL. Factors for Differential Outcome Across Cancers in Clinical Molecule-Targeted Fluorescence Imaging. J Nucl Med. 2022 Nov;63(11):1693-1700. doi: 10.2967/jnumed.121.263674. Epub 2022 Mar 24. PMID: 35332092; PMCID: PMC9635681.
30. Erber R, Spoerl S, Mamilos A, Krupar R, Hartmann A, Ruebner M, Taxis J, Wittenberg M, Reichert TE, Spanier G, Spoerl S. Impact of Spatially Heterogeneous Trop- 2 Expression on Prognosis in Oral Squamous Cell Carcinoma. Int J Mol Sci. 2021 Dec 22;23(1):87. doi: 10.3390/ijms23010087. PMID: 35008509; PMCID: PMC8745008.
31. Yang W, Lin Z, Li L, Zhang G. [Expression of VEGF, EGFR, p16 in lip cancers and oral squamous cell carcinomas and their clinic significance]. Zhonghua Kou Qiang Yi Xue Za Zhi. 2002 Mar;37(2):99-101. Chinese. PMID: 12475418.

No immunohistochemistry (n = 14) [1-14]**:**

1. Bagan JV, Mata-Roig M, Cortio-Gimeno J, Murillo-Cortes J, Hens-Aumente E, Poveda-Roda R, Bagan L. Epidermal growth factor receptor copy number in potentially malignant oral disorders and oral squamous cell carcinoma: a short communication and preliminary study. J Oral Pathol Med. 2012 Oct;41(9):662-6. doi: 10.1111/j.1600-0714.2012.01137.x. Epub 2012 Mar 14. PMID: 22417006.
2. Boldrup L, Coates PJ, Hedberg Y, Sjöström B, Dahlqvist A, Nylander K. Expression of p63, COX-2, EGFR and beta-catenin in smokers and patients with squamous cell carcinoma of the head and neck reveal variations in non-neoplastic tissue and no obvious changes in smokers. Int J Oncol. 2005 Dec;27(6):1661-7. PMID: 16273222.
3. Chen IH, Chang JT, Liao CT, Wang HM, Hsieh LL, Cheng AJ. Prognostic significance of EGFR and Her-2 in oral cavity cancer in betel quid prevalent area cancer prognosis. Br J Cancer. 2003 Aug 18;89(4):681-6. doi: 10.1038/sj.bjc.6601171. PMID: 12915878; PMCID: PMC2376917.
4. Gokhale AS, Haddad RI, Cavacini LA, Wirth L, Weeks L, Hallar M, Faucher J, Posner MR. Serum concentrations of interleukin-8, vascular endothelial growth factor, and epidermal growth factor receptor in patients with squamous cell cancer of the head and neck. Oral Oncol. 2005 Jan;41(1):70-6. doi: 10.1016/j.oraloncology.2004.06.005. PMID: 15598588.
5. Hsieh CH, Chang JW, Hsieh JJ, Hsu T, Huang SF, Liao CT, Wang HM. Epidermal growth factor receptor mutations in patients with oral cavity cancer in a betel nut chewing-prevalent area. Head Neck. 2011 Dec;33(12):1758-64. doi: 10.1002/hed.21665. Epub 2011 Jan 31. PMID: 21284055.
6. Jiang Y, Li Y, Ge H, Wu Y, Zhang Y, Guo S, Zhang P, Cheng J, Wang Y. Identification of an autophagy-related prognostic signature in head and neck squamous cell carcinoma. J Oral Pathol Med. 2021 Nov;50(10):1040-1049. doi: 10.1111/jop.13231. Epub 2021 Aug 18. PMID: 34358365.

7. Lo WY, Wang HJ, Chiu CW, Chen SF. miR-27b-regulated TCTP as a novel plasma biomarker for oral cancer: from quantitative proteomics to post- transcriptional study. J Proteomics. 2012 Dec 21;77:154-66. doi: 10.1016/j.jprot.2012.07.039. Epub 2012 Aug 9. PMID: 22902387.
8. Michikawa C, Uzawa N, Sato H, Ohyama Y, Okada N, Amagasa T. Epidermal growth factor receptor gene copy number aberration at the primary tumour is significantly associated with extracapsular spread in oral cancer. Br J Cancer. 2011 Mar 1;104(5):850-5. doi: 10.1038/bjc.2011.22. Epub 2011 Feb 8. PMID: 21304522; PMCID: PMC3048213.
9. Myo K. [Prediction of occult cervical lymph node metastasis in patients with stage I -II squamous cell carcinoma of the oral cavity]. Kokubyo Gakkai Zasshi. 2006 Mar;73(1):100-6. Japanese. doi: 10.5357/koubyou.73.100. PMID: 16629476.
10. Yamada T, Tsuda M, Ohba Y, Kawaguchi H, Totsuka Y, Shindoh M. PTHrP promotes malignancy of human oral cancer cell downstream of the EGFR signaling. Biochem Biophys Res Commun. 2008 Apr 11;368(3):575-81. doi: 10.1016/j.bbrc.2008.01.121. Epub 2008 Feb 6. PMID: 18261460.
11. Zanotti L, Paderno A, Piazza C, Pagan E, Bignotti E, Romani C, Bandiera E, Calza S, Del Bon F, Nicolai P, Ravaggi A. Epidermal growth factor receptor detection in serum and saliva as a diagnostic and prognostic tool in oral cancer. Laryngoscope. 2017 Nov;127(11):E408-E414. doi: 10.1002/lary.26797. Epub 2017 Aug 7. PMID: 28782189.
12. Freier K, Schwaenen C, Sticht C, Flechtenmacher C, Mühling J, Hofele C, Radlwimmer B, Lichter P, Joos S. Recurrent FGFR1 amplification and high FGFR1 protein expression in oral squamous cell carcinoma (OSCC). Oral Oncol. 2007 Jan;43(1):60-6. doi: 10.1016/j.oraloncology.2006.01.005. Epub 2006 Jun 27. PMID: 16807070.
13. Lemos-González Y, Páez de la Cadena M, Rodríguez-Berrocal FJ, Rodríguez-Piñeiro AM, Pallas E, Valverde D. Absence of activating mutations in the EGFR kinase domain in Spanish head and neck cancer patients. Tumour Biol. 2007;28(5):273-9. doi: 10.1159/000110425. Epub 2007 Oct 26. PMID: 17962724.
14. van Keulen S, Nishio N, Birkeland A, Fakurnejad S, Martin B, Forouzanfar T, Cunanan K, Colevas AD, S van den Berg N, Rosenthal E. The Sentinel Margin: Intraoperative *Ex Vivo* Specimen Mapping Using Relative Fluorescence Intensity. Clin Cancer Res. 2019 Aug 1;25(15):4656-4662. doi: 10.1158/1078-0432.CCR-19-0319. Epub 2019 May 29. PMID: 31142505; PMCID: PMC7021202.

Overlaping population (n = 9) [1-9]**:**

1. Ibrahim SO, Vasstrand EN, Liavaag PG, Johannessen AC, Lillehaug JR. Expression of c-erbB proto-oncogene family members in squamous cell carcinoma of the head and neck. Anticancer Res. 1997 Nov-Dec;17(6D):4539-46. PMID: 9494565.
2. Bernardes VF, Gleber-Netto FO, Sousa SF, Silva TA, Aguiar MC. Clinical significance of EGFR, Her-2 and EGF in oral squamous cell carcinoma: a case control study. J Exp Clin Cancer Res. 2010 Apr 29;29(1):40. doi: 10.1186/1756-9966-29-40. PMID: 20429940; PMCID: PMC2877010.
3. Huang SF, Cheng SD, Chien HT, Liao CT, Chen IH, Wang HM, Chuang WY, Wang CY, Hsieh LL. Relationship between epidermal growth factor receptor gene copy number and protein expression in oral cavity squamous cell carcinoma. Oral Oncol. 2012 Jan;48(1):67-72. doi: 10.1016/j.oraloncology.2011.06.511. Epub 2011 Aug 9. PMID: 21831696.
4. Huang SF, Chuang WY, Chen IH, Liao CT, Wang HM, Hsieh LL. EGFR protein overexpression and mutation in areca quid-associated oral cavity squamous cell carcinoma in Taiwan. Head Neck. 2009 Aug;31(8):1068-77. doi: 10.1002/hed.21067. PMID: 19360742.
5. Monteiro LS, Diniz-Freitas M, Garcia-Caballero T, Forteza J, Fraga M. EGFR and Ki-67 expression in oral squamous cell carcinoma using tissue microarray technology. J Oral Pathol

Med. 2010 Aug 1;39(7):571-8. doi: 10.1111/j.1600-0714.2009.00876.x. Epub 2010 Feb 14. PMID: 20202087.
6. Monteiro LS, Diniz-Freitas M, Warnakulasuriya S, Garcia-Caballero T, Forteza J, Fraga M. An immunohistochemical score to predict the outcome for oral squamous cell carcinoma. J Oral Pathol Med. 2018 Apr;47(4):375-381. doi: 10.1111/jop.12682. Epub 2018 Feb 10. PMID: 29344992.
7. Shah NG, Trivedi TI, Tankshali RA, Goswami JA, Shah JS, Jetly DH, Kobawala TP, Patel KC, Shukla SN, Shah PM, Verma RJ. Molecular alterations in oral carcinogenesis: significant risk predictors in malignant transformation and tumor progression. Int J Biol Markers. 2007 Apr-Jun;22(2):132-43. doi: 10.1177/172460080702200207. PMID: 17549669.
8. Verma J, Dhingra V, Srivastava S, Misra V, Varma K, Singh S. Evaluation of epidermal growth factor receptor expression by a new scoring system in head-and-neck squamous cell carcinoma and its association with various pathological prognostic factors. J Oral Maxillofac Pathol. 2018 Jan-Apr;22(1):11-17. doi: 10.4103/jomfp.JOMFP_137_16. PMID: 29731551; PMCID: PMC5917517.
9. Trivedi TI, Tankshali RA, Goswami JV, Shukla SN, Shah PM, Shah NG. Identification of site-specific prognostic biomarkers in patients with oral squamous cell carcinoma. Neoplasma. 2011;58(3):217-26. doi: 10.4149/neo_2011_03_217. PMID: 21391738.

Review (n = 2) [1,2]**:**

1. Niculescu Talpoş IC, Rumel RC, Scurtu AD, Dinu Ş, Miron MI, Preduţ AD, Cosoroabă RM, Popovici RA, Raica M. Oral squamous cell carcinomas: a histopathological review of multiple cases from Western Romania. Rom J Morphol Embryol. 2021 Oct-Dec;62(4):929-937. doi: 10.47162/RJME.62.4.05. PMID: 35673812; PMCID: PMC9289717.
2. O-charoenrat P, Rhys-Evans PH, Modjtahedi H, Eccles SA. The role of c-erbB receptors and ligands in head and neck squamous cell carcinoma. Oral Oncol. 2002 Oct;38(7):627-40. doi: 10.1016/s1368-8375(02)00029-5. PMID: 12167415.

In vitro (n = 1) [1]**:**

1. Arany I, Chen SH, Megyesi JK, Adler-Storthz K, Chen Z, Rajaraman S, Ember IA, Tyring SK, Brysk MM. Differentiation-dependent expression of signal transducers and activators of transcription (STATs) might modify responses to growth factors in the cancers of the head and neck. Cancer Lett. 2003 Sep 10;199(1):83-9. doi: 10.1016/s0304-3835(03)00345-8. PMID: 12963127.
